# Large-scale functional assessment of variants of the potassium channel Kir2.1: Clinical and comparative insights

**DOI:** 10.1016/j.jbc.2025.110985

**Published:** 2025-11-26

**Authors:** Corey L. Anderson, Saba Munuwar, Janay K. Walters, Seamus F. McWilliams, Molly Melnick, Emma R. Langer, Igor Bereslavskyy, Maxwell R. Milaitis, Louise Reilly, Lee L. Eckhardt

**Affiliations:** 1 Department of Medicine, University of Wisconsin-Madison, Madison, Wisconsin, USA; 2 Cellular and Molecular Arrhythmias Research Program, University of Wisconsin-Madison, Madison, Wisconsin, USA

**Keywords:** KIR2.1, variant of uncertain significance, potassium channel, protein stability, yeast assay, deep mutational scan

## Abstract

Hundreds of *KCNJ2* (Kir2.1) variants of uncertain significance (VUS) have been associated with Andersen-Tawil Syndrome (ATS). Remarkably, most Kir2.1 variants' surface expression and function have been described *via* deep mutational scans (DMS). These results have provided an unprecedented picture of Kir2.1 structure-function relationships and insights into VUS. However, these studies are limited by the lack of robust validation. We performed a flow cytometry-based Kir2.1 surface expression assay for 70 variants (61 ATS-linked) distributed across the potassium inward rectifier channel, a thermal stability assay of 20 variants with reduced surface expression, a yeast-based functional assay for 20 variants (10 pathogenic or likely pathogenic (P/LP) and 10 VUS), as well as whole-cell patch clamp for 13 variants (4 P/LP and 9 VUS). Kir2.1 cell surface expression results showed that ∼30% of variants have reduced surface expression when co-expressed with WT, and that ∼25% disagreed with the DMS datasets. ∼70% of variants with reduced surface tested had reduced thermal stability. Our yeast assay showed all 10 P/LP variants exhibiting LOF, and 7 out of 10 VUS were LOF, in contrast to the DMS method. Patch clamp data further validated the yeast assay. Our data underscores the limitations to interpreting the Kir2.1 DMS datasets, demonstrates a proof-of-principle yeast assay as a complementary method to better inform ClinVar classifications, and provides several lines of evidence for LOF of 9 Kir2.1 VUS in the process.

The finding of variants of uncertain significance (VUS) negatively impacts clinical management of suspected genetically associated conditions, including inherited arrhythmias (1). One major class of proteins linked to inherited arrhythmias are ion channels, which orchestrate the flow of ions across the sarcolemma in response to stimuli to generate cardiac action potentials. The macroscopic current generated across a group of ion channels depends on the number of channels at the surface, open probability, and single-channel current. Mutations can perturb one or more of these properties in a variety of ways, leading to disease, such as misfolding and defective trafficking, disrupting ligand binding or disrupting the selectivity filter, respectively (2, 3). Functional analyses targeting one or more of these LOF mechanisms can provide strong evidence for or against variant pathogenicity per the ACSM guidelines in combination with other criteria (4). The strength of support, however, can be improved with proper validation studies and multiple lines of functional evidence (4, 5). Given the expanding number of VUS, deep mutational scans (DMS), also known as multiplex assays of variant effects or MAVEs, have been developed to assess thousands of variants simultaneously, including many proteins identified in inherited arrhythmias (1, 6).

*KCNJ2*, which encodes Kir2.1 and is the dominant protein subunit for the inward rectifying current IK1 sets cardiac resting membrane potential and completes terminal repolarization of cardiac cells (Fig. 1). Mutations in *KCNJ2* are associated with several sudden death syndromes including Andersen-Tawil Syndrome which is loss-of-function (LOF) and Short QT Syndrome which is gain-of-function (GOF). There have been two DMS for *KCNJ2*, the first of which characterized nearly all possible variants' surface expression and function for ∼90% of the amino acids providing unprecedented insights into structure-function relationships (7). Unfortunately, validation for this study was relatively limited with no supporting patch clamp data and moderate correlations to published surface (8) and fitness (9) scores (ρ = 0.72–0.75) reported. Furthermore, the voltage sensor dye used had low dynamic range making the functional assay less robust than the surface data. Further, a mouse Kir2.1 sequence was used as well as only homomeric mutant Kir2.1 channels, which is not physiologically ideal particularly as ATS is an autosomal dominant disease where WT-mutant subunit interactions may alter channel trafficking and function (8, 10, 11, 12). It remains unclear how many mutant channels are needed for LOF for each mutation but a study of D71Y supports two mutant channels being required (13) while only one T74A mutant was required in a separate study (14). An additional DMS study of Kir2.1 surface expression covered the entire channel and included deletions and insertions (15), but has the same limitations described above.

**Figure 1:**
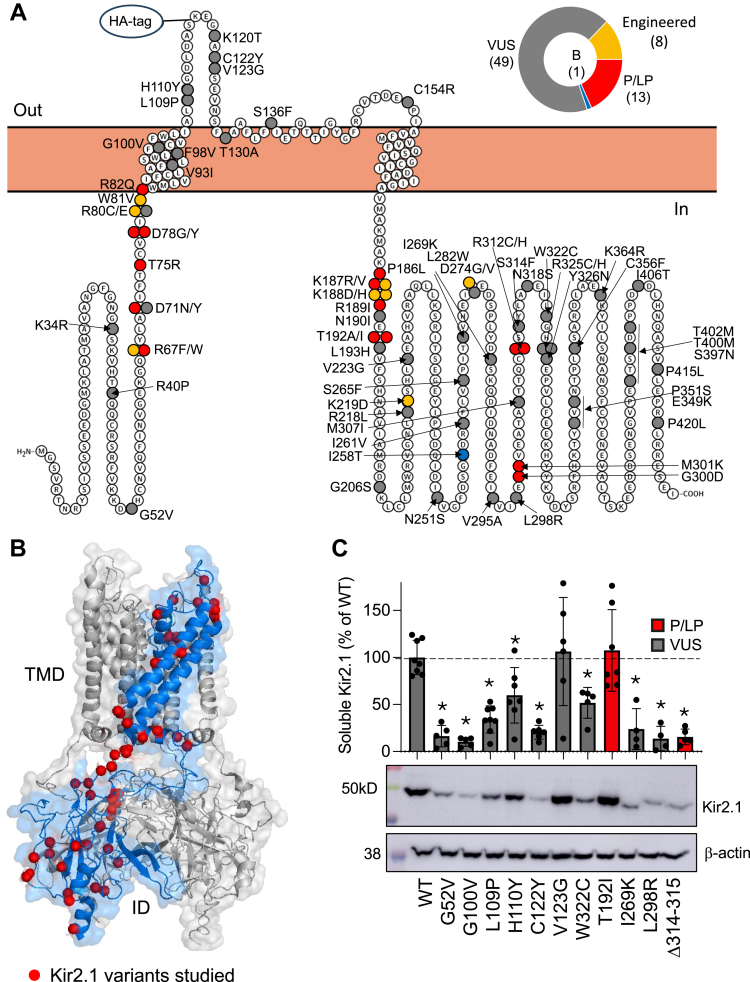
**Properties of Kir2.1 variants studied.***A*, primary structure showing each variant color-coded based on ClinVar classifications of pathogenic/likely pathogenic (P/LP, *red*), benign (*blue*), variant of uncertain significance (VUS, *gray*) or not ATS-associated or engineered (*yellow*). *B*, tertiary structure with location of each variant shown with a *red ball* on one subunit. *C*, representative western blot of several variants showing Kir2.1 variant expression of correct size and expression levels relative to WT. One-way ANOVA revealed a significant difference between groups (F (20,82) = 10.15, *p* < 0.001). ∗*p* < 0.05 compared to WT according to Dunnett's multiple comparison *post hoc* tests. All variants in Table S4 were included for comparisons. Transmembrane domain (TMD), intracellular domain (ID). P/LP in *red* and VUS *gray*.

Due to the noted limitations of prior work and the importance of variant classification for clinical management, we sought conclusive validation to better inform clinicians and scientists in identifying structure/function relationships and pathogenicity assessment per ACMG Guidelines (4, 5). In doing so, we co-expressed 70 Kir2.1 variants with WT and found ∼30% have reduced surface expression; most of which were a consequence of Kir2.1 destabilization determined by a thermal stability assay. Surprisingly, ∼25% showed different levels of discordance with the Kir2.1 DMS results. A similar level of disagreement was also found between the DMS functional data and predicted LOF mutations from updated P/LP classifications in ClinVar. We then tested an alternative yeast-based functional assay for 20 Kir2.1 variants, which performed better than the voltage-sensitive dye assay when compared to P/LP classification and patch clamp data for 13 variants. This study shows conflicting results with the DMS datasets and presents an alternative or complementary functional assay amenable for DMS. We also provide surface expression data for 70 Kir2.1 variants, thermal stability data for 20 variants and experimental supporting LOF evidence for 9 VUS.

## Results

### Kir2.1 variant selection and expression model

We performed a large-scale surface expression analysis of 70 *KCNJ2*-missense variants spread across the Kir2.1 potassium channel (16) (Fig. 1, *A* and *B*). 48 of these we classified as VUS from Clinvar (includes VUS, conflicting, or not provided descriptors) or described elsewhere, 13 are pathogenic or likely pathogenic, 1 is benign and an additional 9 engineered variants not disease associated (Fig. 1, Table S1). Using the population control database GenomAD ver 4.1 (17), 43 of these variants are absent from the population. Of the 28 listed, most are at low frequency (∼10^−7^) including some classified as P/LP (R67W, R82Q, R312C, R312H), with several at a substantially higher frequency ∼10^−4^ (*e.g.* V93I, E349K, N410S, and P415L) (Table S2). We then performed site-directed mutagenesis (see Table S3 for list of primers) to generate a cDNA library containing all 70 variants into Kir2.1 modified to contain an extracellular HA tag. Figure S1 shows human and mouse sequence alignment and location of extracellular tags (18). Western blots of lysates from transiently transfected HEK 293 cells for each variant all expressed at the expected size of 48kD shown in Figure S2. Interestingly, many variants had differences in detergent-soluble expression levels with 20 quantified in Table S4 and representative examples shown in Figure 1*C*. To rule out differences in plasmid quality, four different plasmid preps that underwent several freeze–thaw cycles were compared with a fresh prep, and expression levels were largely uniform (100 ± 18; n = 9) and shown in Figure S2.

**Figure 2:**
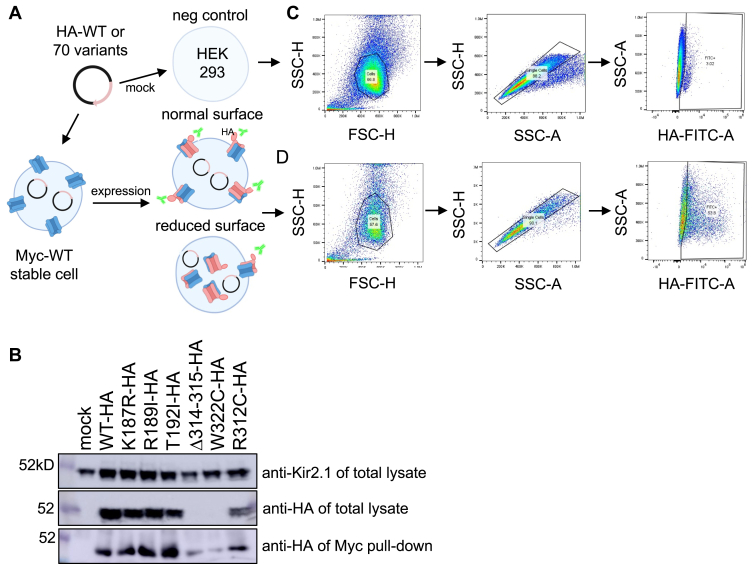
**Kir2.1 WT and variant co-expression and flow cytometry assay.***A*, HEK 293 overexpression model for flow cytometry showing HA-tagged Kir2.1 expression in a MYC-tagged Kir2.1 stable cell line. *B*, Pull-down assay demonstrating HA-tagged variants co-assembling with Myc-tagged WT. *Top blot* shows Kir2.1 antibody detecting HA-Kir2.1 and Myc-Kir2.1 from stable cell line. *Middl*e *blot* shows detection of transfected HA-Kir2.1 variants. *Bottom blot* shows detection of all HA-Kir2.1 variants pulled-down from Myc beads. Uncropped western blots are shown in Figure S2. *C*, flow cytometry gating for untransfected HEK 293 negative control cell population followed by single cell gating and FITC gating for negative/positive cells. *D*, same gating used as (*C*) for each variant population to determine relative signal compared to WT.

Since ATS is inherited in an autosomal dominant fashion, we overexpressed each HA-tagged Kir2.1 variant (HA-Kir2.1) in a HEK 293 cell line stably expressing MYC-tagged WT Kir2.1 (Myc-Kir2.1) Figure 2*A* (19). To show that HA-Kir2.1 channels co-assembled with the MYC-Kir2.1 channels, we performed a pulldown assay for WT and 24 variants, with 6 shown in Figure 2*B* (see Fig. S3 for uncropped blots and an additional 17 variants). All HA-Kir2.1 variants were able to co-assemble with MYC-Kir2.1, including ATS-linked W322 C and Δ314 to 315, which disturbs Kir2.1 tetrameric assembly (8). Other, low-abundant variants also co-assembled with WT (*e.g.* I269K and L298R), showing that co-assembly for even unstable, trafficking defective mutations is detectable in our co-expression system. To get a sense of WT: variant stoichiometry, we estimate that transiently transfected variants express ∼2-fold over the WT stable cell line. Figure S3*A*, which shows intensity levels of transfected Kir2.1 (variant and WT) about twice the mock transfected (WT only) determined by densitometry. However, only ∼50% of cells are transfected from flow data in Figure 2, *C* and *D*, supporting a 2:1 variant to WT expression model. We used this heteromeric expression model favoring 2 or more variant subunits in tetrameric Kir2.1 for surface expression to better mimic the autosomal dominant ATS disease, compared to the homomeric channel expression models used in the DMS (7, 15).

### Kir2.1 variant surface expression

**Figure 3:**
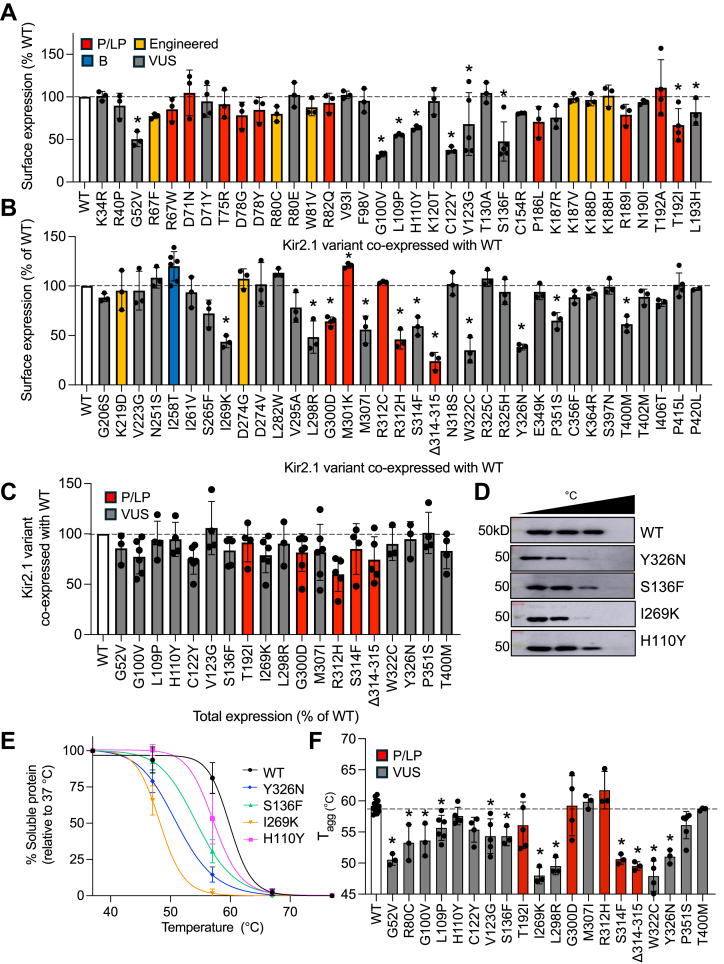
**Kir2.1 variant surface expression.** Surface expression for each Kir2.1 variant as a percentage of WT surface expression split into *panel* (*A*) and (*B*). Variants with ClinVar classifications of P/LP in *red*, benign in *blue*, VUS in *gray* or engineered in *yellow*. One-way ANOVA of all variants in *panel* (*A*) and (*B*) revealed a significant difference between groups (F (70,157) = 8.595, *p* < 0.001). ∗*p* < 0.05 compared to WT according to Dunnett's multiple comparison *post hoc* tests. *C*, total expression determined by flow cytometry for variants found to be reduced in Fig. *A* and *B* as a percentage of WT total expression. One-way ANOVA analysis revealed no significant differences between groups. *D*, representative western blots of cellular thermal shift assays for WT and four variants which were quantified to determine T_agg_ in (*E*). (*F*) T_agg_ values for all variants shown compared to WT. One-way ANOVA revealed a significant difference between groups (F (23,76) = 13.33, *p* < 0.001). ∗*p* < 0.05 compared to WT according to Dunnett's multiple comparison *post hoc* tests. P/LP in *red* and VUS *gray*.

To determine surface expression, we over-expressed each HA-Kir2.1 variant in the Myc-Kir2.1 stable cell line and measured surface expression compared to WT only using flow cytometry (Fig. 2, *C* and *D*). Of the 70 variants measured (n ≥ 3), we found 20 with reduced surface expression and only M301K with increased expression (*p* < 0.05) shown in Figure 3, *A* and *B*. By classification, five variants were pathogenic or likely pathogenic T192I, G300D, M301K, R312H, Δ314 to 315, and the other 15 were VUS (G52V, G100V, L109P, H110Y, C122Y, V123G, S136F, L193H, I269K, L298R, M307I, S314F, W322C, Y326N, P351S, and T400M). Several other mutations also show slightly reduced surface expression (*e.g.* R67W, D78G, C154R, S265F) but did not reach statistical significance. Loss of surface expression was also consistent with other reports of C122Y (20) and W322C (21), for example.

Next, to determine if the reduced surface expression was due to decreased total expression, we performed flow cytometry detecting total HA-Kir2.1 protein for 19 of these variants (n ≥ 3) and found that while many showed a slight reduction (*e.g.* R312H), none were statistically different from WT (Fig. 3*C*). To better visualize how this data compares to the surface data, we compared surface and total expression together in Figure S4. While some of the reduction in surface expression is from less total expression, some other mechanism accounts for the differences, like intracellular retention from loss of stability or misfolding, which is supported by our western blots of detergent soluble protein shown in Figure 1*C*. We then performed western blots to quantify the detergent-soluble Kir2.1 for all variants (n ≥ 3) and found 15 with reduced solubility, shown in Table S4.

### Thermal stability of Kir2.1 variants with reduced surface expression

To follow up on our solubility results, we directly measured the stability of all variants with reduced surface expression using a cellular thermal shift assay (CETSA) recently applied to another potassium channel, Kv7.1 (22). CETSA was performed on 19 Kir2.1 variants (n ≥ 3) transiently transfected in HEK 293 cells to determine the midpoint temperature where half of the protein has aggregated (T_agg_) over a range of temperatures by measuring western blot intensity of the soluble lysate for each temperature compared to 37 °C reference. Figure 3*D* shows example western blots for four variants quantified to determine T_agg_ values shown in Figure 3*E* Uncropped western blot examples for these and all variants are shown in Figure S5 and T_agg_ values for all variants are shown in Figure 3*F* and Table S4. We found 12 of the variants have reduced thermal stability (*p* < 0.05), which largely correlate with the solubility results (Table S4). The exceptions were H110Y, C122Y, M307I, and R312H, which had normal thermal stability but reduced solubility and V123G, which had reduced thermal stability but normal solubility. This data also suggests that testing NP40 detergent solubility might be a simple first approach to determining if a Kir2.1 variant is destabilizing.

### Surface and functional validation of deep mutational scans

**Figure 4:**
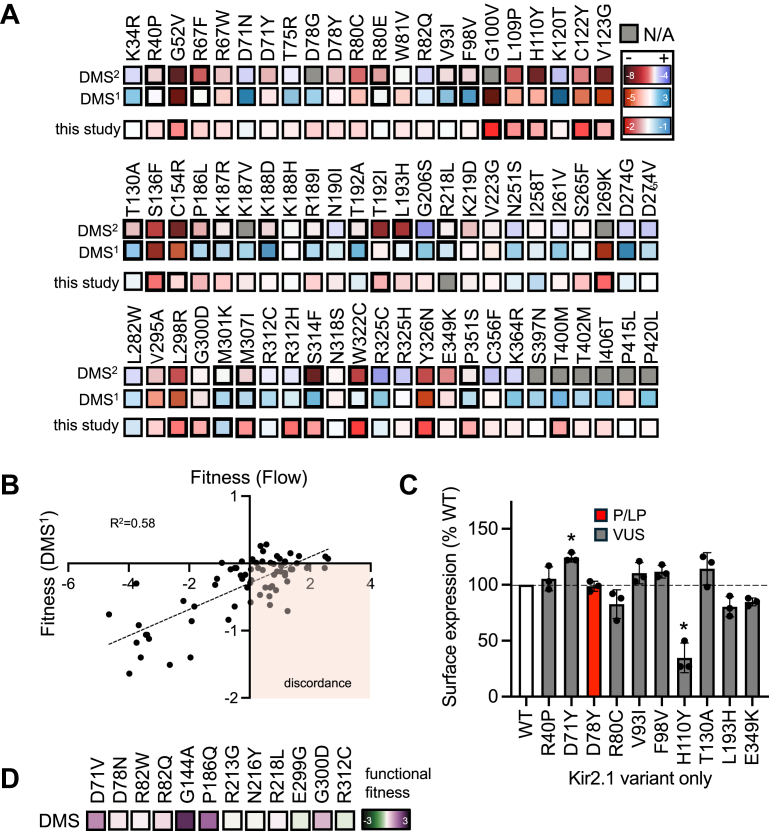
**Heatmap data for two reported DMSs and our surface expression results.***A*, heatmap colors were copied from two published heatmaps (DMS^1^ and DMS^2^) and generated from our own surface expression values (Table S2) for this study. DMS^2^ colors were inversed for easier comparison. Boldened boxes in the DMS heatmaps indicate conflicting results between the two DMSs. Boldened boxes in this study indicate statistically significant reductions in surface expression (see Fig. 3, *A* and *B* and Table S2). *Gray boxes* indicate data not available (N/A). *B*, linear regression analysis for DMS^1^ fitness scores vs flow fitness scores (log2 transformed relative to WT) listed in Table S2. *C*, mean surface expression levels for select variants in (*A*) expressed alone as homomeric variant channels for a direct comparison to the DMS surface data. One-way ANOVA revealed a significant difference between groups (F (10, 22) = 21.65, *p* < 0.001). ∗*p* < 0.05 compared to WT according to Dunnett's multiple comparison *post hoc* tests. P/LP in *red* and VUS *gray.D*, 12 of 47 variants labelled P/LP in ClinVar that show normal or GOF in DMS^2^ heatmap. DMS^1^ from Macdonald, *et al.* 2023 (5) and DMS^2^ from Coyote-Maestas *et al.*, 2022 (6).

Since two surface expression DMS have been reported for Kir2.1 with little to no comparison or validation studies, we first compared the two heatmap results for 70 variants characterized in this study. Remarkably, we found 18 variants (∼25%) that showed some level of disagreement between the studies highlighted in Figure 4*A* with boldened squares. For example, some variants showed a slight loss (R40P) or gain (T192A) of surface expression in one DMS and none in the other, while other variants were more striking, with S314F showing LOF in DMS^2^ and increased surface expression in DMS^1^.

Next, to systematically assess whether the two assays differ in their estimates of variant effects, we log2-transformed all 70 variant surface expression results to match the fitness scores of DMS^1^ obtained from MAVEDB database (23) shown in Table S2 (DMS^2^ unavailable). A linear regression was fitted in Figure 4*B*, which shows a moderate positive correlation (Pearson R^2^ = 0.58) between the two datasets. However, many variants deviate, highlighting systematic differences with ∼20/70 (∼30%) of variants falling into quadrant IV with opposing fitness scores. The DMS has a larger dynamic range and may thus overestimate the changes seen compared to the flow-based method or *vice versa*.

To determine if these results were due to the different expression models used (*i.e.* heteromeric Kir2.1 channels in this study vs. homomeric channels in DMS), we repeated surface flow cytometry for 9 of these variants expressed alone as homomeric variant channels (n = 3). Figure 4*C* and Table S1 show that homomeric expression did not significantly affect any of the variants with near normal surface expression as heteromeric channels. However, H110Y, which was 64 ± 3% of WT when expressed as a heteromeric channel (Fig. 3*A*) reduced further to 35 ± 13% as a homomeric channel, showing that some variants exert a dominant-negative effect on surface expression. In this context, it shows that WT subunits can influence surface expression of variants, highlighting the importance of the heteromeric model even though differences other than tetrameric stoichiometry likely account for much of the conflicting results with the DMS data.

Finally, to assess the validity of the Kir2.1 DMS that examined function *via* changes in membrane potential, we identified all ClinVar *KCNJ2* variants that were labeled P or LP and found 48, which are listed in Table S5. Of these, 2 were GOF associated with SQT3 (D172N and M301K), and 2 had stress-induced ventricular arrhythmic manifestations (R82W and P186Q) but were reported as LOF *via* a dominant-negative effect (24). Excluding the SQT3 mutations, these 46 variants should exhibit LOF in the DMS (see Table S5). However, 12 (26%) were found to have near normal function or GOF as shown in Figure 4*D*. Combined, these comparisons show substantial conflicting results between the two DMS studies, our surface expression results with DMS results and DMS functional data with P/LP classifications (see Table S5).

### Yeast-based functional analysis of Kir2.1 variants

**Figure 5:**
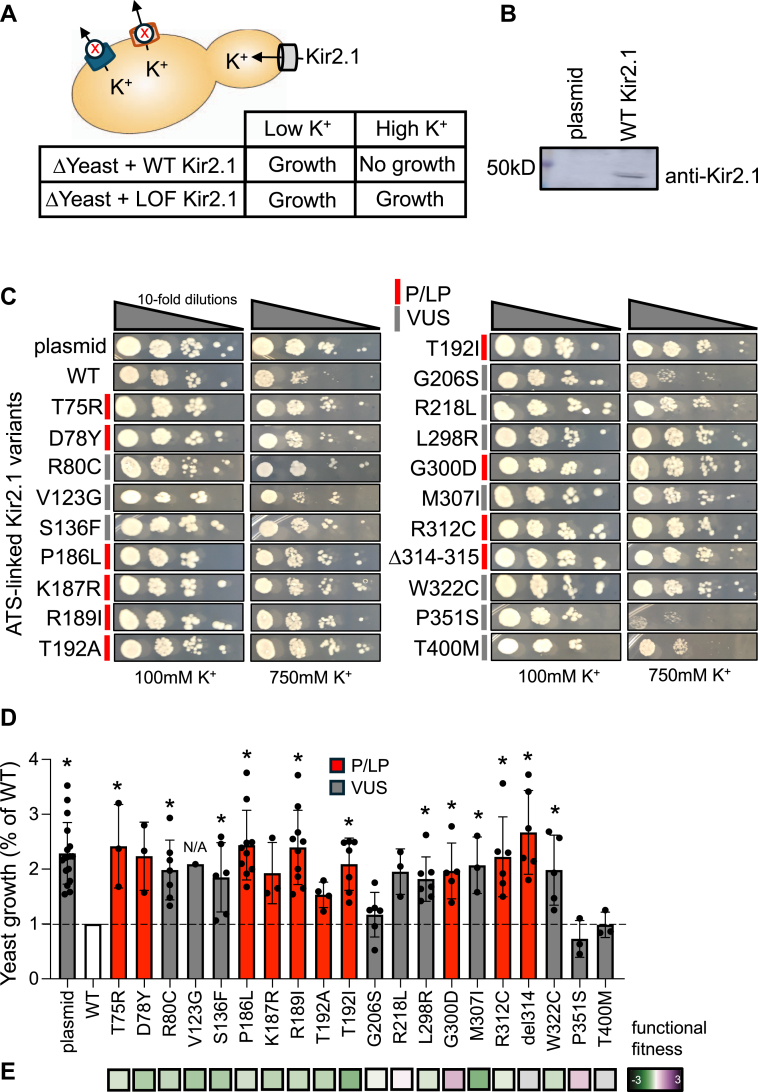
**Yeast assay.***A*, illustration of the yeast growth assay where the B31 yeast strain lacks the ability to export potassium making high potassium concentrations too toxic for growth when expressing functional Kir2.1 in contrast to LOF variants. *B*, western blot of B31 yeast lysates showing expression of WT at correct size compared to plasmid control with more examples shown in Figure S6. *C*, yeast growth for Kir2.1 variants plated at 10-fold serial dilutions on plates at 100 mM and 800 mM potassium. P/LP are color-coded *red* and VUS *gray*. *D*, comparison of yeast growth relative to WT for plasmid and all variants shown in (*C*). One-way ANOVA revealed a significant difference between groups (F (20,125) = 6.421, *p* < 0.001). ∗*p* < 0.05 compared to WT according to Dunnett's multiple comparison *post hoc* tests. *E*, functional heatmaps from DMS^2^ showing LOF (*green*) to GOF (*purple*) for each variant. P/LP in *red* and VUS *gray*.

Since there was a surprisingly large disagreement with our Kir2.1 variant surface expression results and P/LP classifications with the DMS datasets, we assessed an alternative, yeast-based functional assay that could be made multiplexable to compare to the DMS dataset and P/LP classifications. Figure 5*A* shows a schematic of a *Saccharomyces cerevisiae* strain deficient in K^+^ efflux (“B31”) previously described, where growth is inhibited under high K^+^ concentrations when functional Kir2.1 channels are expressed, in contrast to LOF variants, where growth is restored from lack or diminished K^+^ influx (25). This assay has only been reported for a few variants (26), and we expand on these here with an additional 20 variants as validation and a proof-of-principle. To first establish the assay conditions, we tested expression of several Kir2.1 variants in B31 yeast cells by western blot to show that it runs at the expected size of 48kD shown in Figure 5*B*. Uncropped western blot and other examples are shown in Figure S6, *A* and *B*. Next, we overexpressed 6 P/LP LOF variants and one VUS in addition to WT and a plasmid-only control on selective agar plates at four different K^+^ concentrations (0 mM, 200 mM, 400 mM, and 800 mM) to identify growth inhibition. We found WT growth inhibition starting around 400 mM compared to the LOF variants, which grew normally (Fig. S6*C*). We then compared the growth of these same variants and 8 more (10 P/LP and 10 VUS total) variants to WT Kir2.1 at 100 mM and 750 mM K^+^ to further validate this assay at higher concentrations to show maximal effects ideally suited for any future DMS analysis. Figure 5*C* shows that all 10 P/LP variants showed normal growth similar to the plasmid control compared to WT, supporting these variants as LOF. This included pathogenic G300D, R312C, and VUS R218L, shown to be LOF (27), in contrast to the DMS dataset showing normal function (Fig. 4*D*). Additionally, 7 of the 10 VUS tested showed normal growth, indicating LOF. In contrast, G206S, P351S and T400M showed substantially reduced growth, suggesting Kir2.1 functional channel expression similar to WT.

To help visualize variant differences better, we performed densitometry to quantify yeast growth (see Fig. S6*D* for examples), shown in Figure 5*D*. All variants showed an increase in growth (*i.e.* LOF) except G206S, P351S, and T400M. D78Y, K187R, T192A, and R218L, while not statistically significant, were also increased. The functional heatmap for DMS^2^ is also shown below for comparison in Figure 5*E*, revealing good agreement overall but key differences with LOF R218L, G300D and perhaps R312C, which are benign in the DMS. These results also provide experimental evidence for 7 VUS being dysfunctional, supporting a P/LP classification.

### Patch clamp functional analysis of Kir2.1 variants

**Figure 6:**
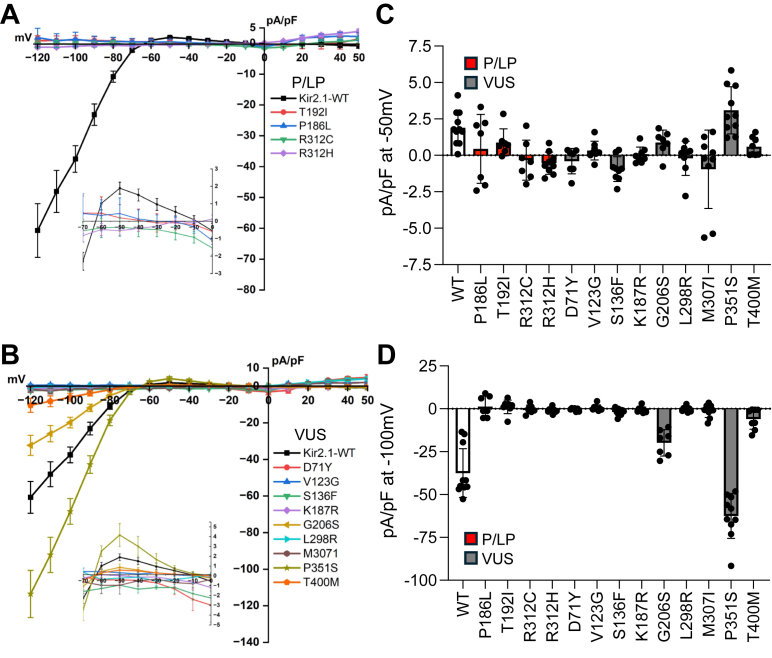
**Whole cell patch clamp of ATS-linked Kir2.1 variants.***A*, I-V plots for 4 variants listed as P/LP in ClinVar. *B*, I-V plots for 9 variants listed as VUS in ClinVar. *C*, mean outward current at −50 mV ± SD. *D*, mean inward current at −100 mV ± SD. P/LP in *red* and VUS *gray*.

Patch-clamp recording is the gold standard technique for determining K^+^ ion channel function, with automated approaches increasingly being used to rapidly screen ion channel VUS (6, 28). To help validate our yeast assay results, we generated 13 stable cell lines in HEK 293 cells expressing four homomeric channel variants listed as P/LP (P186L, T192I, R312C, and R312H) and nine variants listed as VUS (representative western blot for WT and several VUS shown in Fig. S2*G*) and performed whole-cell patch-clamp (n ≥ 7). For the four P/LP variants shown in Figure 6*A*, none showed outward (Fig. 6*C*) or inward (Fig. 6*D*) current consistent with LOF. For the nine LOF variants shown in Figure 6*B* D71Y, V123G, S136F, K187R, L298R, and M307I showed no current, while G206S, P351S and T400M exhibited various levels of outward current (Fig. 6*C*) with inward rectification (Fig. 6*D*) (see also Table S6). Additionally, G300D and R218L also exhibit no current (27) in contrast to the DMS (Fig. 4*D*). Inward currents for the VUS were all statistically different from WT, for example, but since stable cell lines can have variable current densities between clones, direct comparisons could be misleading, and so these results are described only qualitatively. However, only G206S, P351S and T400M exhibit outward current with inward rectification characteristic of a functional inward rectifying channel. These results are also consistent with P/LP classifications and the yeast assay results. Combined, these results provide strong experimental evidence for 9 VUS being LOF (D71Y, R80C, V123, S136F, K187R, R218L, L298R, M307I, and W322C), while G206S, P351S and T400M do not. Furthermore, genomAD analysis, a database for reporting variant allele frequencies, also supports these conclusions with LOF alleles being absent from the general population while G206S, P351S and T400 M alleles being present; the latter two having much higher frequencies (see Table S2).

## Discussion

The most unexpected result of this study was the ∼25% discordance between our surface expression results and the reported DMS (7, 15) given that essentially the same flow cytometry-based assay was used. This lack of agreement does not seem due to our WT-variant co-expression model in contrast to the DMS variant-only model. We found that variants with reduced surface expression in the DMS that were normal in our co-expression study remained normal when expressed as homomeric channels. While homomeric vs. heteromeric expression might explain some of the differences as reported for T75M for example (10), other factors likely account for most including 1) mouse Kir2.1 was used in the DMS, which contains a 6 amino acid and one gap difference from human Kir2.1 which could affect trafficking (29), 2) stable cell lines expressing from a single genomic locus were used in the DMS compared to expression from an episomal plasmid during transient transfection in our study, 3) DMS used a FLAG tag (DYKDDD) in contrast to our HA tag (YPYDVPDYA) tag inserted at the same site and 4) method of quantification in the DMS is more complicated with multiple steps (*i.e.* sorting, next-generation sequencing, fitness scoring) potentially introducing more room for errors (30). For most variants however, perhaps the simplest explanation could be that the cut-off stringency in DMS is too low for determining whether a variant has reduced surface expression as a LOF mechanism in that model with such a large dynamic range. In our study, several variants trend low but are not statistically significant, yet are described as reduced in the DMS (7) where only slight changes in surface expression are shown (*e.g.* R40P, R80C, V93I, *etc.*). Further, many variants in the DMS show increased surface expression, which would be GOF; a mechanism underlying SQTS unless there was a concomitant reduction in Kir2.1 current. However, SQTS is extremely rare and these were all LOF ATS variants. We only found a significant increase in surface expression for SQTS M301K. Regardless, our results suggest that impaired trafficking might not be as common of a LOF mechanism for ATS-linked Kir2.1 variants as reported (7, 15, 31). Our results also underscore how conflicting data from different experimental models can hinder variant classification (∼10% of KCNJ2 missense variants have conflicting classification in ClinVar) and the importance of more than one assay to test variant function along with expert curation (4, 5, 32). In conflicting cases, a more physiologically relevant model may be required to assess surface expression and pathogenicity of ion channelopathies. For example, variability in surface expression results of the long QT type 2 associated Kv11.1 H70R was assessed using induced pluripotent stem cell-derived cardiomyocytes (iPSC-CMs) (33). While a stable cell line over-expressing Kv11.1 H70R showed normal trafficking (34), a transient transfection method led to reduced surface expression (35), which was further supported by an iPSC-CM model (33).

The discordance in functional results is less surprising given the limitations to the DMS method used (*i.e.* poor resolution of the voltage-sensitive dye) and acknowledged by the authors (7). While the DMS structure-function relationship interpretations are informative in a broader context, our study suggests that the DMS datasets may not be as strong for variant classification nor pathogenic mechanism in many cases. We show that the yeast assay may be a more robust method for functionally assessing Kir2.1, which should also be amenable to a DMS where yeast cells expressing a variant library could be grown under low and high [K+] to generate fitness scores. Yeast have also proven to be a useful tool for studying potassium channels (36) including inward rectifier pharmacology (37), trafficking (12), and function (25, 26, 38). Yeast have also been utilized for a DMS of calmodulin associated with inherited arrhythmias (39).

Our study also underscores the importance of proper validation studies and may have broader implications to DMS; particularly using flow cytometry to assess surface expression. To highlight some other DMS, ∼220 mutations covering 11 residues of the Kv11.1 S5 helix (40) and ∼1400 mutations covering 75 residues of the Kv11.1 PAS domain (28) have been characterized. Robust validation studies are somewhat lacking in the former study with only 5 variants characterized by imaging and patch-clamp analysis. However, the latter study performed automated patch-clamp for over 400 variants showing strong correlations between peak tail density and trafficking efficiency (ρ = 0.77) and was consistent with a previously reported immunoblot-based surface assay (ρ = 0.93) (35) and PAS domain stability assay (ρ = 0.80) (41). Remarkably, a DMS for the IKs subunit *KCNE1* also characterized nearly all *KCNE1* variants' surface expression and function. Functionally, only a moderate correlation was shown compared to patch-clamp analysis of 71 variants performed or reported (ρ = −0.64) (42). Last, a triple drug, cell survival-based assay was developed to study the functional effects of 248 *SCN5A* variants in the voltage sensor of Na_v_1.5 (43) and patch-clamp validated 8 of 9 (88%). This relatively small error rate however could mischaracterize a substantial number of the >700 Na_v_1.5 VUS if applied comprehensively.

Like the DMS, our study also comes with similar limitations. Heterologous overexpression models in non-native cell types might exaggerate expression levels and miss proteins important in channel surface expression, pitfalls that can be overcome in iPSC-CMs as demonstrated by a multiplexed assessment of *MYH7* missense VUS (44). High overexpression models may also lead to non-physiological effects as seen with our western blot expression data where a significant number of variants formed detergent insoluble aggregates from misfolding. Misfolding-induced loss of surface expression also underlies many other potassium channels including Kv1.2, (45), Kv7.1 (22), and Kv11.1 (41). It remains to be seen if this phenomenon occurs in non-overexpressing cell models. Interestingly, some of these variants were stable with normal expression levels suggesting alternative mechanisms of Kir2.1 retention. For example, variants might not cause Kir2.1 misfolding but result in local domain destabilization or disrupt interactions required for forward trafficking triggering quality control mechanisms (8, 46). Our results also suggests that a western blot analysis of detergent insoluble Kir2.1 might be a relatively quick way to identify destabilizing LOF variants; an approach we demonstrated for several soluble domains of other large multi-domain proteins including Kv11.1 (41). Transient transfection may also be an inferior model to the stable cell lines used in the DMS. These methods could be compared to iPSC-CMs for example. Also, we only used homomeric mutant channels for our patch clamp and yeast assay for direct comparison to the DMS, but each could be modified to co-express WT in future studies. Nevertheless, DMS will continue to rapidly advance our understanding of protein structure-function relationships, channelopathies and other cardiac arrhythmia syndromes (1). Validation studies and alternative methodologies as highlighted in this study can reveal conflicting results but also help support pathogenicity assessment in most cases. We propose conflicting results can then be addressed individually with more robust functional assays such as whole-cell patch-clamp (1, 6, 47) and iPSC-CMs (33, 48) to better inform pathogenicity.

## Conclusion

Our study highlights strengths and underscores limitations to interpreting Kir2.1 DMS datasets. We found variability in assay performance and promote that external validation of these broad datasets is imperative for clinical variant curation. Our data provides several lines of evidence for LOF of 9 Kir2.1 labeled as VUS in ClinVar. Finally, we demonstrate a proof-of-principle yeast assay as complementary method to better inform variant classifications.

## Experimental procedures

### Variant selection and mutagenesis

Andersen-Tawil Syndrome-associated *KCNJ2* variants were mostly identified and classified using the ClinVar database (https://www.ncbi.nlm.nih.gov/clinvar). The genomAD (ver 4.1) database was used to find allele frequencies of variants in the general population. All missense variants were made using the QuikChange II XL kit (Agilent) using primers designed by Integrated DNA Technologies using our Kir2.1 and/or HA-tagged Kir2.1 pcDNA3 plasmids as templates (see Table S3 for list of primers). Restriction digest analysis was used to test the integrity of all variant constructs, and the coding region was Sanger sequenced at the UW-Biotechnology Center. These constructs were used for HEK 293 expression. For yeast expression, the coding sequence for each variant was subcloned into the pYES2 plasmid (kindly provided by Dr Sojin Shikano at University of Illinois at Chicago) using the EcoRI restriction site and correct orientation determined by Sanger sequencing. Several plasmids were also fully sequenced by Plasmidsaurus using Oxford Nanopore Technology for quality control.

### HEK 293 Cell Culture

Human Embryonic Kidney cells (HEK 293 from ATCC) were maintained in DMEM containing 1 g/L glucose, 1 mM sodium pyruvate, 4 mM L-glutamine and 10% FBS in a humified incubator at 37 °C with 5% CO2. An equal number of HEK 293 cells alone or stably expressing MYC-tagged WT Kir2.1 were plated in 12 well plates and grown to 80 to 90% confluency before being transfected with 1 μg of HA-Kir2.1 variant cDNA using a DNA/Lipofectamine 2000 (Qiagen) ratio of 1:3. Media was changed the next day and 48 h after transfection, cells were harvested for western blot, pulldown assay, or flow cytometry. For stable cell line generation, transfected cells were selected for in media containing geneticin, diluted to new plates to isolate single colonies and expression of each clone screened by western blot followed by whole-cell patch-clamp.

### Yeast assay

The B31 yeast strain and pYES2-hKir2.1 plasmid were kindly provided by Dr Sojin Shikano at University of Illinois at Chicago. The B31 yeast strain was streaked and grown on YPD plates (1% yeast extract, 2% bactopeptone, 2% glucose, 120 mg/ml adenine hemisulfate, 1% agar) at 30 °C for use in experiments. B31 overnight growths from a single colony were then used to transform each plasmid using the Quick & Easy Yeast Transformation Mix (Takara Bio USA, Inc). Transformed cells were plated on YNB selective media (6.7 g/L yeast nitrogen base without amino acids (Sigma), 2% glucose, 10 mg/ml adenine hemisulfate and 0.73 g/L methionine-and uracil-dropout amino acid mixture (United States Biological) supplemented with leucine, 25 mM Tris-HCL pH 7.0, and 1% agar. For growth assays, single colonies for each variant were grown in YNB media overnight and then diluted to an OD_600_ of 0.1. 10-fold serial dilutions were then made and 2 μl of each dilution were plated on YNB plates supplemented with varying K+ concentrations and grown at 30 °C for 48 h before imaging.

### Western blot

For Kir2.1 expression in HEK 293 cells, an equal number of cells were lysed in HEK 293 lysis buffer (50 mM Tris-HCl pH 7.4, 150 mM NaCl, 1% NP40 and HALT protease inhibitor (Thermo Scientific). For Kir2.1 expression in B31 yeast cells, an overnight growth from a single colony was diluted in 3 ml to OD_600_ 0.3 and shook at 30 °C for 4 h. Cells were pelleted and lysed in 100uL CelLytic Y Lysis Reagent (Sigma) with HALT protease inhibitor. For both lysates, insoluble material was spun down at 15,000*g* for 10 min and supernatants were mixed with an equal amount of modified Laemmli buffer (125 mM Tris, pH 6.8, 4% SDS, 8 M urea, 20% glycerol, 200 mM DTT, and 0.02% pyronin Y). Proteins were separated by SDS PAGE on a 4 to 15% gradient gel, transferred to PVDF membranes, blocked in 5% dry milk and 0.1% Tween-20 in PBS and detected with the following antibodies: 1:1000 anti-GFP-HRP, 1:1000 mouse anti-MYC-HRP, 1:1000 mouse anti-HA, 1:2000 rabbit anti-Kir2.1, 1:1000 mouse anti HA all from Santa Cruz Biotechnology. 1:10,000 donkey anti-rabbit or mouse conjugated to HRP were also used for signal detection where appropriate.

### Pull-down assay

12 well plates of Myc-WT Kir2.1 stable cells were transfected with 1 μg of HA-WT, each HA-Kir2.1 variant, or mock control and expressed for 48 h. Cells were lysed in 350 μl NP40 lysis buffer and 20 μl was added to 20 μl modified Laemmli buffer for total protein sample. The remainder was spun down at 15,000*g* for 10 to remove insoluble debris and 20uL of supernatant added to modified Laemmli buffer for soluble protein sample. The remaining soluble fraction was incubated with prewashed Myc-agarose (Thermo Scientific) and rotated overnight at 4 °C. Myc-agarose was then washed in NP40 buffer and eluted in equal amounts of NP40 lysis buffer and modified Laemmli buffer and boiled for 5 min. Myc-agarose was then spun down and eluted proteins along with total and soluble samples were analyzed by western blot as described above.

### Flow cytometry

An equal number of transfected cells from a 12 well plate were dissociated to single cells with 0.25% Trypsin-EDTA at 37 °C for 5 min and then pelleted at 1000 rpm for 5 min. For total internal labeling, supernatant was removed, and cells were fixed in 1% paraformaldehyde at 37 °C for 10 min in the dark, pelleted, and then resuspended in ice-cold 90% methanol for 30 min. Cells were then pelleted and washed with 3 ml FACS+ buffer (DPBS without Ca^2+^/Mg^2+^, 0.5% bovine serum albumin, 0.1% NaN_3,_ 0.1% Triton X-100) to remove methanol, pelleted again and resuspended in 80 μl FACS+ buffer containing HA-FITC antibody. Samples were incubated at room temperature in the dark for 30 min, washed with 3 ml FACS+ buffer and resuspended in 500 μl FACS+ buffer for analysis. For surface labeling, supernatant was removed, and cells were incubated with 80uL FACS- buffer (FACS+ buffer without 1% Triton X-100) containing HA-FITC antibody for 30 min at room temperature in the dark. Cells were then fixed by directly adding 1% paraformaldehyde at 37 °C for 10 min in the dark. Fixative was washed out with 3 ml of FACS- buffer and resuspended in 500 μl FACS- buffer for analysis. For negative control cells, identical methods were used with mock transfected cells. Data were collected on an Attune Nxt flow cytometer (Thermo Scientific) and analyzed with FlowJo.

### Cellular thermal stability assay (CETSA)

HEK 293 cells were transiently transfected with 1 μg of WT and each variant in 12 well plates. The following day, cells were washed with PBS and resuspended in PBS containing HALT protease inhibitor (Thermo Scientific). An equal volume of uniformly mixed cells was transferred to a PCR strip tube and heated in PCR cycler from 37 °C, to 77 °C in 10 °C increments for 3 min each followed by immediate lysis with an equal volume of HEK 293 lysis buffer described above. Insoluble aggregates were spun down at 15,000*g* for 10 min and supernatants were mixed with an equal amount of modified Laemmli buffer and ran on western blot as described above. The signal intensity of each variant band on western blot was normalized to the signal at 37 °C. Curves were fit to a four-parameter symmetric sigmoidal curve to determine T_agg_ in GraphPad 10.0.

### Electrophysiology

HEK 293 cells were stably transfected Kir2.1-WT and homomeric mutants subset including (D71Y, V123G, S136F, P186L, K187R, T192I, G206S, R218L, G300D, L298R, M307I, R312L, R312H, P351S, T400M). Cells were split to 2 ml dishes 24 h prior to whole cell recordings. Electrophysiology experiments were done using an Axopatch 200B amplifier and pClamp 10 (Molecular Devices). Whole cell patch clamp experiments were performed at room temperature. Borosilicate glass capillary patch electrodes with resistance 2 to 4 MΩ when filled (Model P-97, Sutter Instruments). Pipettes were filled with internal solution containing (in mM) K-gluconate 150, EGTA 5, MgATP 5, and HEPES 10 and pH adjusted to 7.2 with KOH. External bath solution for I_K1_ measurements contained (in mM) NaCl 148, KCL 5.4, CaCl_2_ 1.8, MgCl_2_ 1, HEPES 15, NaHPO_4_ 0.4, D-glucose 5.5 and pH adjusted to 7.4 with NaOH. Whole cell patch clamp mode was applied to the cells in the voltage clamp configuration using Axopatch 200B amplifier (Molecular Devices). Current signals were digitized at 10 kHz, filtered at 2 kHz and stored on IBM-Compatible PC interfaced with a Digidata 1440 analogue to digital converter (Molecular Devices). IK1 was recorded starting from a holding potential of −50 mV, voltages were stepped from −120 to +50 mV with a sequential 10 mV steps increase in 100 ms steps. Barium chloride 0.5 mM in bath solution was used to perfuse the cells for minimum of 2 min and I-V protocol was repeated. These recordings were used to subtract the contaminating current from the previously recorded files of the same cells. Capacitance measurements were taken before barium perfusion as previously described (49) and currents were normalized to cell capacitance. Data analysis was performed using pClamp10 and Origin 2020 software (OriginLab Corporation).

### Statistics

All data are presented as mean ± SD of at least 3 biological replicates using separate transfections for HEK 293 studies and separate growths for yeast plating. For all figures, multiple comparisons were made using one-way ANOVA followed by Dunnett's multiple-comparison *post hoc* test. Surface expression data in Figure 3 was split into two groups, but all data were included in ANOVA analysis. Similarly, Figure 1*C* reflects multiple comparisons analysis on all variants reported in Table S4. *p* < 0.05 was considered statistically significant. Statistical analyses were performed using GraphPad Prism 10 Software.

## Data availability

All data supporting the findings of this study are available in the article and supporting information. Additional data are available upon request from the corresponding authors.

## Supporting information

This article contains supporting information including six supplementary tables S1-S6, six supplementary tables S1-S6 and 19 supporting references (3, 7, 10, 13, 15, 21, 50, 51, 52, 53, 54, 55, 56, 57, 58, 59, 60, 61, 62).

## Conflict of interest

The authors declare that they have no conflicts of interest with the contents of this article.
