# Supplementary material for: Large-scale functional assessment of variants of the potassium channel Kir2.1: Clinical and comparative insights
Source: J Biol Chem. 2025 Nov 26;302(1):110985. doi: 10.1016/j.jbc.2025.110985 (PMC12775956; doi:10.1016/j.jbc.2025.110985)
Supplement: Supplementary Figures [file mmc1.pdf]

```
Mouse 1    MGSVRTNRYSIVSSEEDGMKLATMAVANGFGNGKSKVHTRQQCRSRFVKKDGHCNVQFIN  60
Human 1    ............................................................  60

                                                                 ▼
61         VGEKGQRYLADIFTTCVDIRWRWMLVIFCLAFVLSWLFFGCVFWLIALLHGDLDTSKVSK  120
61         ......................................................A..EG.  120

121        ACVSEVNSFTAAFLFSIETQTTIGYGFRCVTDECPIAVFMVVFQSIVGCIIDAFIIGAVM  180
121        ............................................................  180

181        AKMAKPKKRNETLVFSHNAVIAMRDGKLCLMWRVGNLRKSHLVEAHVRAQLLKSRITSEG  240
181        ............................................................  240

241        EYIPLDQIDINVGFDSGIDRIFLVSPITIVHEIDEDSPLYDLSKQDIDNADFEIVVILEG  300
241        ............................................................  300

301        MVEATAMTTQCRSSYLANEILWGHRYEPVLFEEKHYYKVDYSRFHKTYEVPNTPLCSARD  360
301        ............................................................  360

361        LAEKKYILSNANSFCYENEVALTSKEEEEDSENGVPESTSTDSPPGIDLHNQASVPLEPR  420
361        .........................-..D.............T..D..............  419

421        PLRRESEI   428
420        ........   427
```

▼ extracellular tag location

HA: YPYDVPDYA

FLAG: DYKDDD

Figure S1. Kir2.1 sequence alignment. Mouse (uniProt P35561) and human (uniProt P63252) sequence alignment showing location of extracellular tags. Human sequence with HA tag shown in red was used in this study in contrast to FLAG tag mouse sequence for the DMSs.

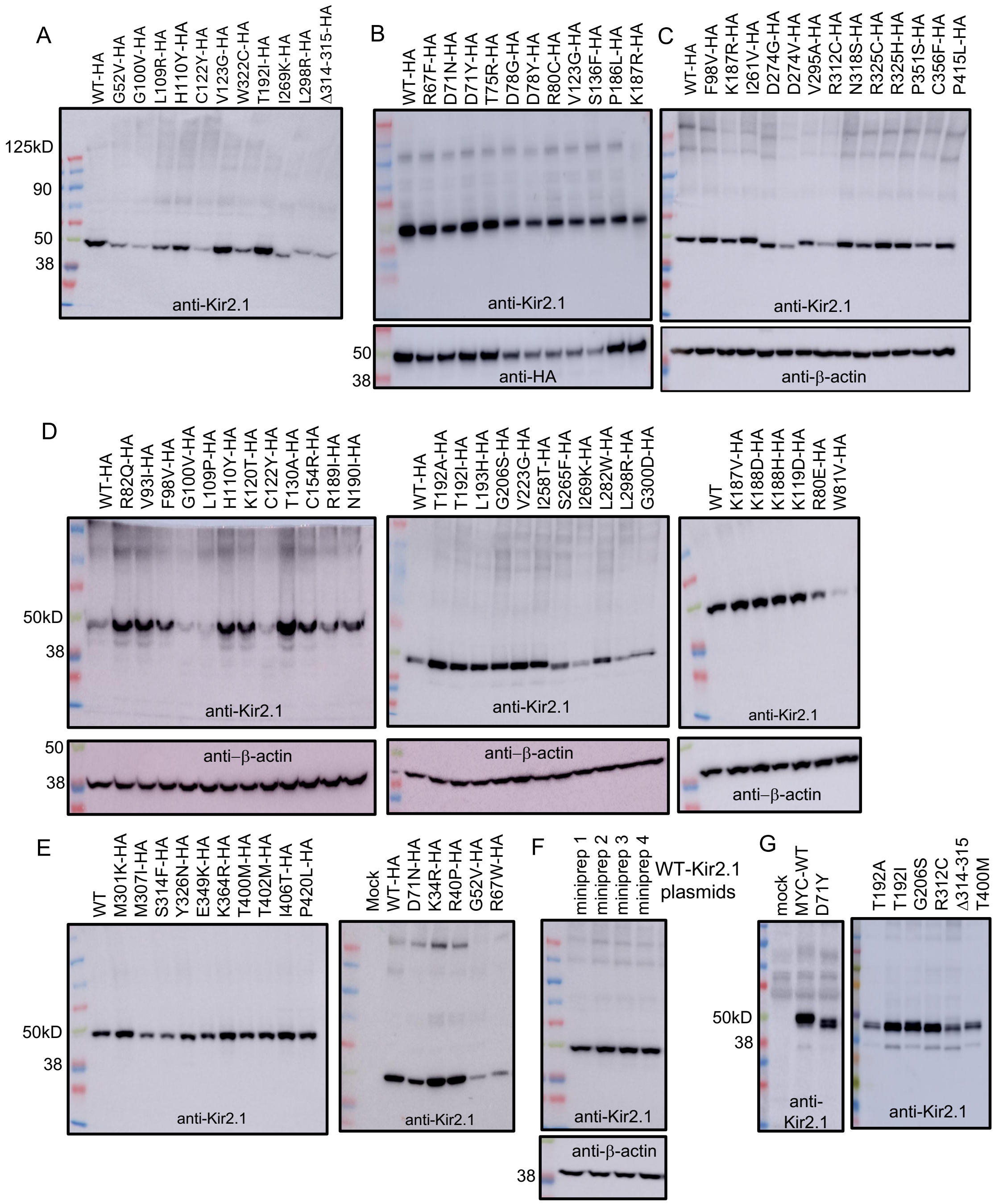

Figure S2. Kir2.1 variant western blots. (A) Uncropped western blot from Fig. 1C showing HA-tagged WT and select variants detected with an anti-Kir2.1 antibody. (**B**) Example western blots of Kir2.1 variants detected with Kir2.1 and HA antibodies. (**C** and **D**) More examples of Kir2.1 variant western blots with β-actin loading control and (**E**) without β-actin loading control to show examples for of all variants studied. (**F**) Western blot examples comparing different WT plasmids with β-actin control**.** (**G**) Western blot examples of stable cell lines used for whole-cell patch clamp data in Fig. 6.

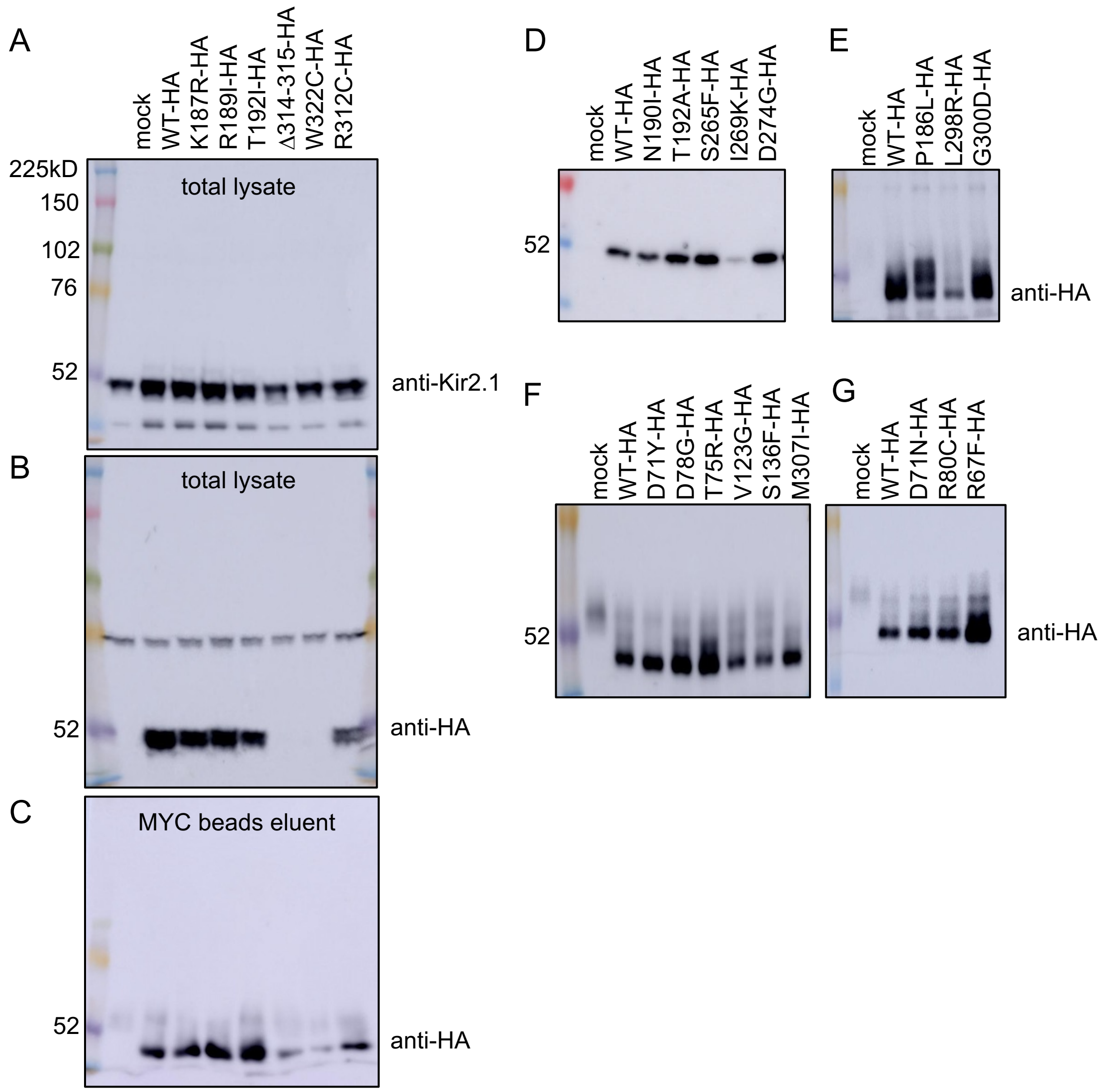

Figure S3. HA-Kir2.1 western blots of MYC-pulldowns. Uncropped western blots from Fig. 2B shown in **A**,**B** and **C**. **D-F** show more sets of HA-tagged variants pulled down with Myc beads indicating WT-variant interactions.

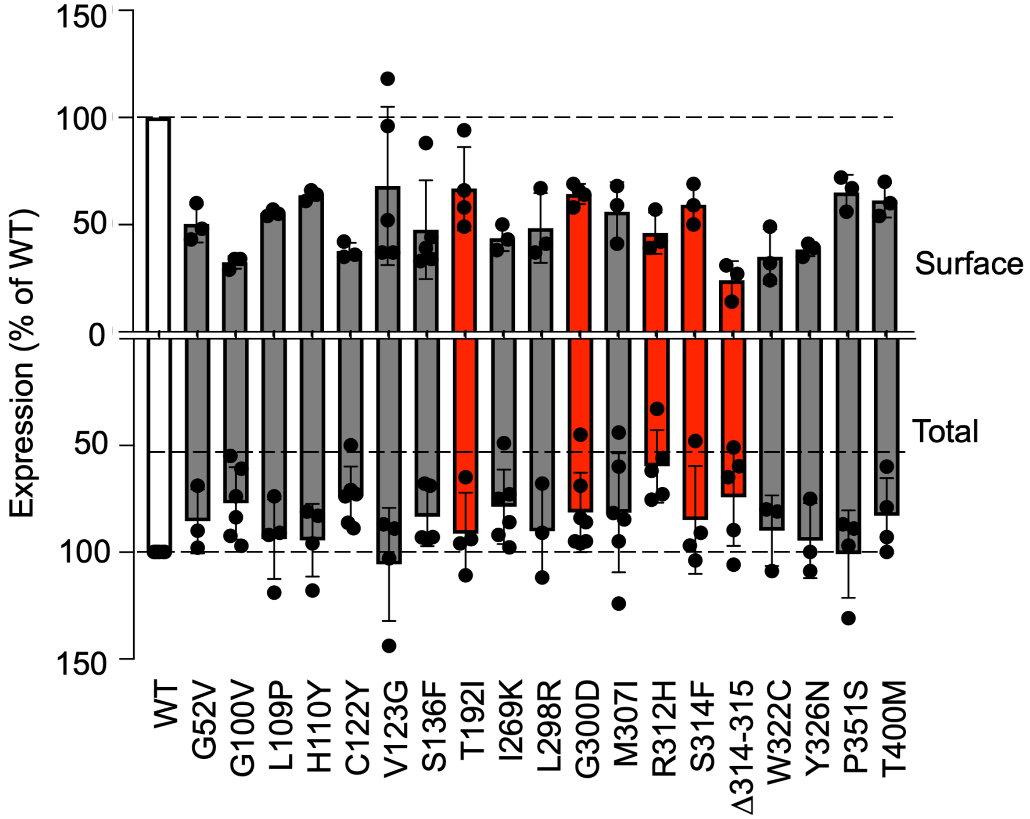

Figure S4. Side-by-side comparison of surface and total expression from flow cytometry data in Fig. 3A-C.

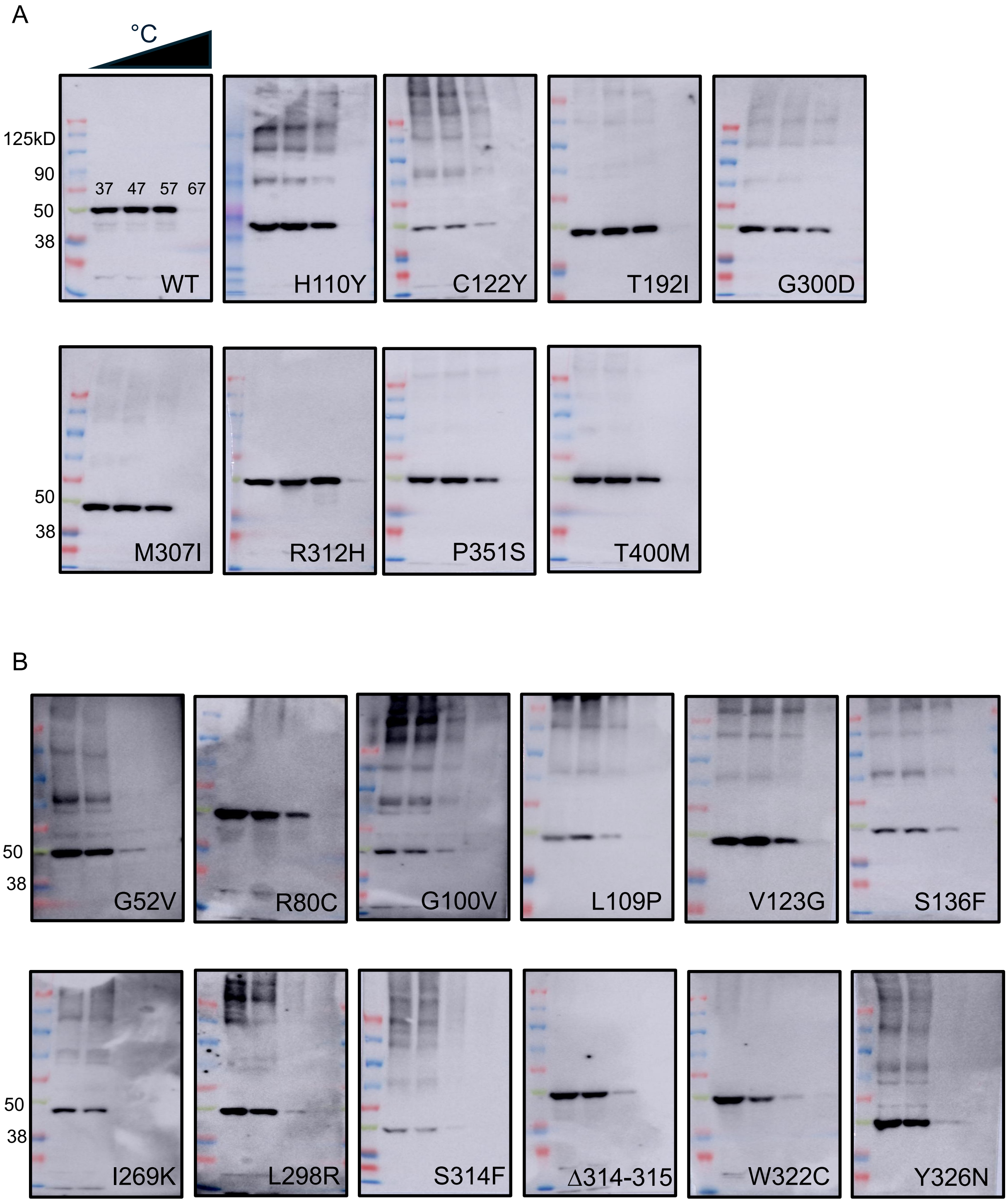

Figure S5. Representative Western blots from CETSA experiments of WT and 20 variants from Fig. 3D-F. Examples of uncropped western blots for variants that were (**A**) thermally stable and (**B**) thermally unstable.

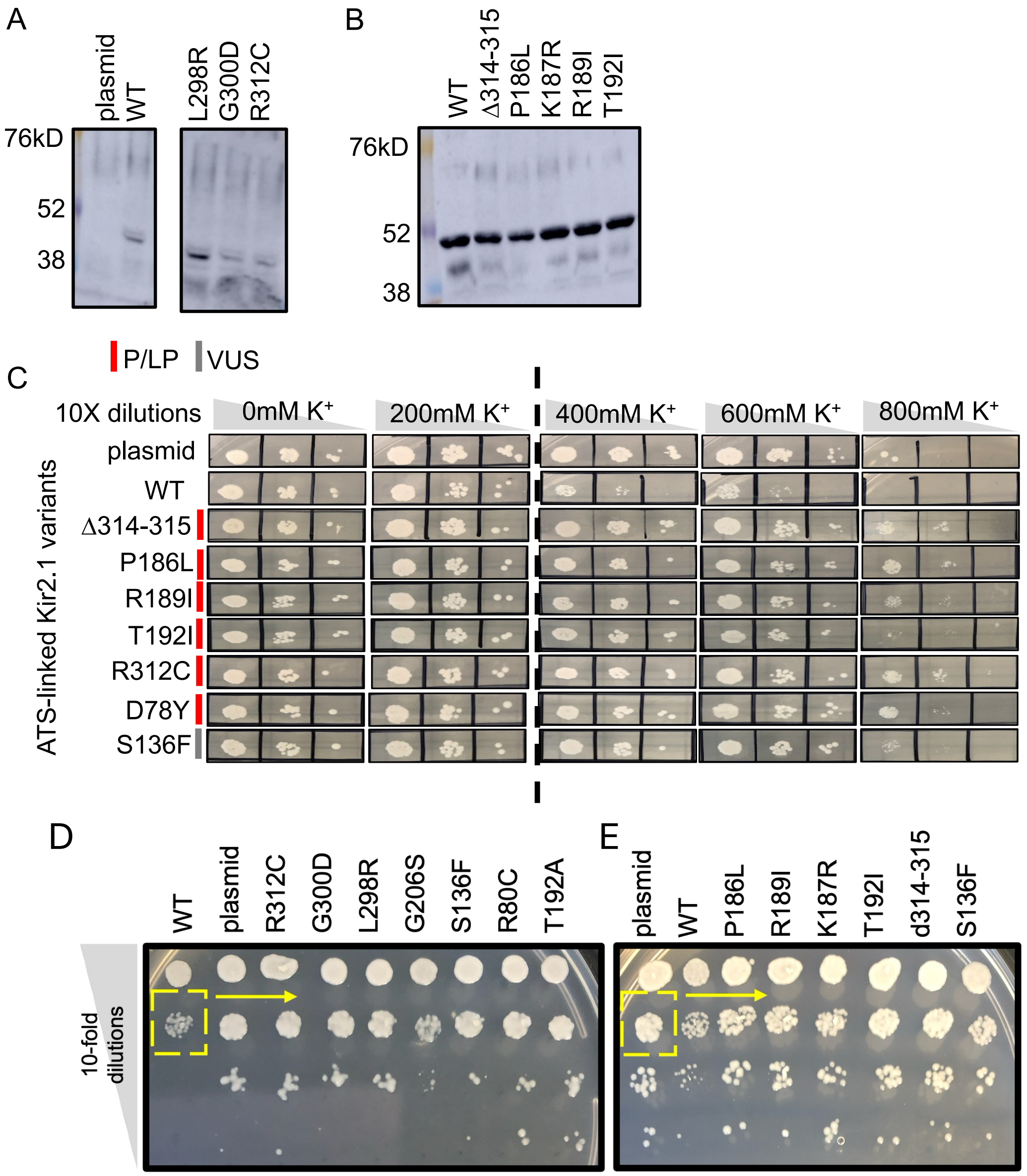

Figure S6. Yeast western blots and plating results. (**A**) Uncropped western blot from Fig 5B. (**B**) More western blot examples of yeast lysates over-expressing Kir2.1 variants running at the expected size of 48kD. (**C**) Yeast growth tests of several Kir2.1 variants plated at 10-fold serial dilutions on plates with different potassium concentrations. Compared to WT, loss-of-function variants exhibit more growth starting at ~400mM K^+ (dashed red line). This difference increases at 600mM and 800mM K^+ where WT cells become too toxic for growth in contrast to the loss-of-function variants. (**D and E**) Examples of yeast plantings used for densitometry. Boxes shown used to quantify yeast density across the first dilution for all data collected. Two examples shown to illustrate variance between experiments with (**E**) showing some growth in the third dilution in contrast to (**D**).
