# Supplementary material for: Large-scale functional assessment of variants of the potassium channel Kir2.1: Clinical and comparative insights
Source: J Biol Chem. 2025 Nov 26;302(1):110985. doi: 10.1016/j.jbc.2025.110985 (PMC12775956; doi:10.1016/j.jbc.2025.110985)
Supplement: Supplementary Tables [file mmc2.pdf]

Kir2.1 variant	ClinVar	DMS surface fitness	This study				DMS function
			Surface (% of WT)	Surface fitness (% of WT)	Variant only surface (% of WT)	Yeast growth (relative to WT)	
**WT**	NA	normal	100	100	100	1	normal
**K34R**	VUS	1.15	101 ± 6 (4)	0.01	NA	NA	slight gain
**R40P**	VUS	0.05	84 ± 15 (3)	-0.25	105 ± 11 (3)	NA	slight gain
**G52V**	VUS	-3.67	53 ± 9 (3)*	-0.92	NA	NA	slight loss
**R67F**	not in ClinVar	-0.08	78 ± 3 (3)	-0.09	NA	NA	slight to no gain
**R67W**	P	-1.08	85 ± 14 (3)	-0.23	NA	NA	slight loss
**D71N**	LP	2.59	105 ± 27 (3)	0.07	NA	NA	slight loss
**D71Y**	VUS[1]	-0.44	95 ± 19 (4)	-0.07	124 ± 4 (3)*	NA	slight loss
**T75R**	P	1.17	91 ± 17 (3)	-0.14	NA	2.4 ± 0.8 ± (3)*	slight loss
**D78G**	P	0.68	78 ± 16 (3)	-0.09	NA	NA	NA
**D78Y**	P	-0.25	85 ± 14 (3)	-0.23	99 ± 5 (3)	1.8 0.8 (3)	slight loss
**R80C**	VUS	-1.14	80 ± 9 (3)	-0.32	83 ± 13 (3)	1.9 ± 0.6 (7)*	slight loss
**R80E**	not in ClinVar	-0.26	102 ± 15 (3)	0.03	NA	NA	slight gain
**W81V**	not in ClinVar	-0.3†	88 ± 9 (3)	-0.18	NA	NA	loss
**R82Q**	P/LP	0.43	93 ± 11 (3)	-0.10	NA	NA	slight gain
**V93I**[a]	conflicting	1.17	102 ± 5 (3)	0.03	110 ± 9 (3)	NA	slight gain
**F98V**	VUS[2]	1.89	95 ± 14 (3)	-0.07	112 ± 6 (3)	NA	slight gain
**G100V**	VUS	-3.98	32 ± 3 (3)*	-1.64	NA	NA	slight gain
**L109P**	VUS	-1.92	55 ± 2 (3)*	-0.86	NA	NA	slight loss
**H110Y**	VUS	-1.83	64 ± 3 (3)*	-0.64	35 ± 13 (3)*	NA	slight loss
**K120T**	conflicting	2.48	95 ± 16 (3)	-0.07	NA	NA	slight gain
**C122Y**	VUS	-1.96	38 ± 4 (3)*	-1.40	NA	NA	slight loss
**V123G**	not provided	-2.19	68 ± 37 (5)*	-0.56	NA	2.1 (1)	slight loss
**T130A**	VUS	0.99	105 ± 12 (3)	0.07	114 ± 14 (3)	NA	slight loss

**S136F**	not provided	-3.39	48 ± 23 (3)*	-1.06	NA	1.9 ± 0.6 (6)*	slight loss
**C154R[b]**	VUS	-3.75	81 ± 1 (3)*	-0.30	NA	NA	slight loss
**P186L**	P	0.65	71 ± 18 (3)	-0.49	NA	2.3 ± 0.7 (10)*	slight loss
**K187R**	VUS[3]	0.87	76 ± 13 (3)	-0.40	NA	1.9 ± 0.6 (3)	slight loss
**K187V**	not in ClinVar	0.9	92 ± 11 (3)	-0.12	NA	NA	slight gain
**K188D**	not in ClinVar	1.91	96 ± 7 (3)	-0.06	NA	NA	loss
**K188H**	not in ClinVar	0.22	101 ± 13 (3)	0.01	NA	NA	slight gain
**R189I**	LP	0.74	79 ± 12 (3)	-0.34	NA	2.4 ± 0.7 (10)*	slight loss
**N190I**	VUS	1.41	94 ± 3 (3)	0.60	NA	NA	slight loss
**T192A**	P	1.41	111 ± 33 (3)	0.15	NA	1.4 ± 0.3 (4)	slight loss
**T192I**	P	0.03	67 ± 19 (4)*	-0.58	NA	2 ± 0.5 (7)*	slight loss
**L193H**	VUS	0.02	82 ± 15 (3)	-0.29	80 ± 9 (3)	NA	slight loss
**G206S**	conflicting	1.17	88 ± 4 (3)	-0.18	NA	1.2 ± 0.3 (6)*	slight loss
**R218L**	conflicting	1.56	NA	NA	NA	2 ± 0.4 (3)*	normal
**K219D**	not in ClinVar	0.28	95 ± 20 (3)	-0.07	NA	NA	loss
**V223G**	VUS	-0.49	95 ± 20 (3)	-0.07	NA	NA	slight loss
**N251S**	VUS	0.9	108 ± 10 (3)	0.11	NA	NA	slight gain
**I258T**	B	0.36	120 ± 15 (6)	0.26	NA	NA	slight gain
**I261V**	VUS	1.36	94 ± 16 (3)	-0.09	NA	NA	slight loss
**S265F**	VUS	-0.34	72 ± 14 (3)	-0.47	NA	NA	slight loss
**I269K**	VUS	-3.74	44 ± 6 (3)*	-1.18	NA	NA	slight loss
**D274G**	not in ClinVar	2.5	107 ± 10 (3)	0.10	NA	NA	slight loss
**D274V**	VUS	0.69	101 ± 22 (3)	0.01	NA	NA	slight loss
**L282W**	conflicting	0.44	113 ± 4 (3)	0.18	NA	NA	slight gain
**V295A**	VUS	0.52	78 ± 15 (3)	-0.26	NA	NA	normal
**L298R**	VUS[3]	-3.43	48 ± 16 (3)*	-1.06	NA	1.7 ± 0.4 (7)*	slight loss

**G300D**	P	-0.5	64 ± 5 (4)*	-0.64	NA	2 ± 0.5 (5)*	slight gain
**M301K**	P	0.55	121 ± 3 (3)	0.28	NA	NA	slight loss
**M307I**	VUS	-0.7	56 ± 14 (3)*	-0.84	NA	2.1 ± 0.5 (3)*	slight loss
**R312C**	P/LP	0.09	104 ± 1 (3)	0.06	NA	2.2 ± 0.7 (6)*	normal
**R312H**	P/LP	-3.33	46 ± 10 (3)*	-1.12	NA	NA	loss
**S314F**	VUS	-4.65	59 ± 10 (3)*	-0.76	NA	NA	normal
**Δ314-315**	P	NA	24 ± 9 (3)*	NA	NA	2.6 ± 0.7 (6)*	NA
**N318S**	VUS	0.42	102 ± 12 (3)	0.03	NA	NA	slight gain
**W322C**	VUS[4]	-2.64	35 ± 13 (3)*	-1.51	NA	1.9 ± 0.6 (5)*	slight loss
**R325C**	conflicting	1.63	108 ± 8 (3)	0.11	NA	NA	slight gain
**R325H**	VUS	0.04	94 ± 13 (3)	-0.09	NA	NA	slight gain
**Y326N**	VUS	-3.6	38 ± 3 (3)*	-1.40	NA	NA	slight loss
**E349K**	conflicting	-0.63	94 ± 8 (4)	-0.09	85 ± 4 (3)	NA	slight to no gain
**P351S**	not provided	0.67	65 ± 8 (3)*	-0.62	NA	0.7 ± 0.3 (3)	slight gain
**C356F**	VUS	-0.15	89 ± 7 (3)	-0.17	NA	NA	slight gain
**K364R**	VUS	1.03	92 ± 5 (3)	-0.12	NA	NA	slight gain
**S397N**	VUS	0.79	99 ± 8 (3)	-0.01	NA	NA	NA
**T400M**	conflicting	1.25	61 ± 8 (3)*	-0.71	NA	1 ± 2 (3)	NA
**T402M**	VUS	0.81	89 ± 8 (3)	-0.17	NA	NA	NA
**I406T**	VUS	1.24	83 ± 4 (3)	-0.27	NA	NA	NA
**P415L**	conflicting	-0.92	101 ± 12 (5)	0.01	NA	NA	NA
**P420L**	conflicting	1.04	97 ± 4 (3)	-0.04	NA	NA	NA

Table S1. Properties of HA variants. Kir2.1 column lists all ATS variants studied as of January 2025 with classifications described in ClinVar column. V93I[a] is linked to Short QT Syndrome. VUS not in ClinVar are indicated with numbers referencing the source. C154R[b] is a novel, unpublished variant identified at the UW Arrhythmias Clinic. "Not in ClinVar" were engineered for other studies. DMS Surface fitness column lists fitness scores obtained from the MAVEDB database (www.mavedb.org) for DMS[1] by MacDonald C.B. et al. 2023[5]. Surface and variant only columns in this study show mean ± SD of each variant's surface and total expression co-expressed with WT or alone relative to WT. Surface fitness scores for this data were surface data converted to log2 fold change fitness scores to compare with DMS fitness scores. Yeast growth are densitometry values relative to WT. DMS function descriptions qualitatively determined from published heatmap data[6]. Asterisks indicate statistical significance ($P < 0.05$) using one-way ANOVA analysis with Dunett's post hoc test. Number of experiments in parentheses.

Variant	ClinVar	genomAD	Allele count
K34R	VUS	2.10E-05	6
R67W	P	6.20E-07	1
R82Q	P/LP	7.90E-07	4
V93I	conflicting	1.13E-04	183
L109P	VUS	6.20E-07	1
K120T	conflicting	3.47E-05	8
G206S	conflicting	1.06E-05	3
N251S	VUS	3.65E-06	9
I261V	VUS	1.06E-05	3
D274G	not in ClinVar	6.20E-07	1
D274V	VUS	6.20E-07	1
L282W	conflicting	1.53E-05	28
R312C	P/LP	1.24E-06	2
R312H	P/LP	6.20E-07	1
N318S	VUS	1.53E-05	29
R325C	conflicting	5.46E-05	94
R325H	VUS	1.24E-06	7
E349K	conflicting	2.88E-04	40
P351S	not provided	1.20E-04	10
C356F	VUS	8.78E-06	20
K364R	VUS	6.20E-07	1
S397N	VUS	6.20E-07	1
T400M	conflicting	5.80E-05	82
T402M	VUS	5.43E-05	76
N410S	conflicting	5.61E-04	445
P415L	conflicting	1.35E-04	191
P420L	conflicting	1.83E-06	38
R422L	VUS	2.80E-07	2

Table S2. Kir2.1 variants present in genomAD (ver4.1) listing their ClinVar classification, frequency and allele number.

Kir2.1 variant	Forward primer	Reverse primer
**K34R**	ggctttgggaacgggaggagtaaagtccacacc	ggtgtggactttactcctcccgttcccaaagcc
**R40P**	gtaaagtccacaccccacaacagtgcaggag	ctcctgcactgttgtggggtgtggactttac
**G52V**	cgctttgtgaagaaagatgtccactgtaatgttcagttc	gaactgaacattacagtggacatctttcttcacaaagcg
**R67F**	atgtgggtgagaaggggcaattctacctcgcagacatcttc	gaagatgtctgcgaggtagaattgccccttctcacccacat
**R67W**	ggtgagaaggggcaatggtacctcgcag	ctgcgaggtaccattgccccttctcacc
**D71N**	ggcaacggtacctcgcaaacatcttcaccacg	cgtggtgaagatgtttgcgaggtaccgttgcc
**D71Y**	ggcaacggtacctcgcatatatcttcaccacgtgtgt	acacacgtggtgaagatatatgcgaggtaccgttgcc
**T75R**	gcagacatcttcaccaggtgtgtggacattcgc	gcgaatgtccacacacctggtgaagatgtctgc
**D78G**	accacgtgtgtgggcattcgctggcgg	ccgccagcgaatgcccacacacgtggt
**D78Y**	cttcaccacgtgtgtgtatattcgctggcggtgga	tccaccgccagcgaatatacacacacgtggtgaag
**R80C**	caccacgtgtgtggacatttgctggcggtg	caccgccagcaaatgtccacacacgtggtg
**R80E**	tcttcaccacgtgtgtggacattgagtggcggtggatg	catccaccgccactcaatgtccacacacgtggtgaaga
**W81V**	gtgtgtggacattcgcgtgcggtggatgctggtt	aaccagcatccaccgcacgcgaatgtccacacac
**R82Q**	gtggacattcgctggcagtggatgctggttatc	gataaccagcatccactgccagcgaatgtccac
**V93I**	cttctgcctggctttcatcctgtcatggctgtt	aacagccatgacaggatgaaagccaggcagaag
**F98V**	tcgtcctgtcatggctggtttttggctgtgtgttt	aaacacacagccaaaaaccagccatgacaggacga
**G100V**	cctgtcatggctgtttttgtctgtgtgttttggttgatag	ctatcaaccaaaacacacagacaaaaacagccatgacagg
**L109P**	gttgatagctctgccccatggggacctgg	ccaggtccccatggggcagagctatcaac
**H110Y**	gttgatagctctgctctatggggacctggatgc	gcatccaggtccccatagagcagagctatcaac
**K120T**	gatgcatccaaagagggcacggcttgtgtgtccgagg	cctcggacacacaagccgtgccctctttggatgcatc
**C122Y**	gcatccaaagagggcaaagcttatgtgtccgaggtc	gacctcggacacataagctttgccctctttggatgc
**V123G**	agggcaaagcttgtgggtccgaggtcaacag	ctgttgacctcggacccacaagctttgccct
**T130A**	aggtcaacagcttcgcggctgccttcctc	gaggaaggcagccgcgaagctgttgacct
**S136F**	gctgccttcctcttcttcattgagacccagaca	tgtctgggtctcaatgaagaagaggaaggcagc
**C154R**	ttcagatgtgtcacggatgaacgcccaattgctg	cagcaattgggcgttcatccgtgacacatctgaa
**P186L**	ggccaagatggcaaagctaaagaagagaaacgaga	tctcgtttctcttctttagctttgccatcttggcc
**K187R**	caagatggcaaagccaaggaagagaaacgagactc	gagtctcgtttctcttccttggctttgccatcttg
**K187V**	ggccaagatggcaaagccagtgaagagaaacgagactctt	aagagtctcgtttctcttcactggctttgccatcttggcc
**K188D**	aagacaagagtctcgtttctatcctttggctttgccatcttgg	ccaagatggcaaagccaaaggatagaaacgagactcttgtctt
**K188H**	ccaagatggcaaagccaaagcatagaaacgagactcttgtctt	aagacaagagtctcgtttctatgctttggctttgccatcttgg
**R189I**	atggcaaagccaaagaagataaacgagactcttgtcttc	gaagacaagagtctcgtttatcttctttggctttgccat
**N190I**	gcaaagccaaagaagagaatcgagactcttgtcttca	tgaagacaagagtctcgattctcttctttggctttgc
**T192A**	ccaaagaagagaaacgaggctcttgtcttcagtcaca	tgtgactgaagacaagagcctcgtttctcttctttgg
**T192I**	agccaaagaagagaaacgagattcttgtcttcagtcac	gtgactgaagacaagaatctcgtttctcttctttggct
**L193H**	agaagagaaacgagactcatgtcttcagtcacaatgc	gcattgtgactgaagacatgagtctcgtttctcttct
**G206S**	tgattgccatgagagacagcaagctgtgtttgatg	catcaaacacagcttgctgtctctcatggcaatca
**R218L**	cgagtgggcaatcttctgaaaagccacttggtg	caccaagtggcttttcagaagattgcccactcg
**K219D**	gagtgggcaatcttcgggatagccacttggtggaagc	gcttccaccaagtggctatcccgaagattgcccactc
**V223G**	cggaaaagccacttgggggaagctcatgttcga	tcgaacatgagcttcccccaagtggcttttccg
**N251S**	tccctctggatcaaatagacatcagtgttgggtttgac	gtcaaacccaacactgatgtctatttgatccagaggga
**I258T**	aatgttgggtttgacagtggaaccgatcgtatatttctgg	ccagaaatatacgatcggttccactgtcaaacccaacatt
**I261V**	acagtggaatcgatcgtgtatttctggtgtcccca	tggggacaccagaaatacacgatcgattccactgt
**S265F**	gaatcgatcgtatatttctggtgttcccaatcactatagtc	gactatagtgattgggaacaccagaaatatacgatcgattc
**I269K**	ctggtgtccccaatcactaaagtccatgaaatagatg	catctatttcatggactttagtgattggggacaccag
**D274G**	caatcactatagtccatgaaataggtgaagacagtcctttatatgattt	aaatcatataaaggactgtcttcacctatttcatggactatagtgattg
**D274V**	caatcactatagtccatgaaatagttgaagacagtcctttatatgattt	aaatcatataaaggactgtcttcaactatttcatggactatagtgattg
**L282W**	atagatgaagacagtcctttatatgattggagtaaacaggacattgac	gtcaatgtcctgtttactccaatcatataaaggactgtcttcatctat
**V295A**	cgcagactttgaaatcgcggtcatactggaaggca	tgccttccagtatgaccgcgatttcaaagtctgcg
**L298R**	tgaaatcgtggtcatacgggaaggcatggtggaag	cttccaccatgccttcccgtatgaccacgatttca
**G300D**	gtggtcatactggaagacatggtggaagccact	agtggcttccaccatgtcttccagtatgaccac
**M301K**	gtggtcatactggaaggcaaggtggaagcca	tggcttccaccttgccttccagtatgaccac
**M307I**	tggaagccactgccataacgacacagtgcc	ggcactgtgtcgttatggcagtggcttcca
**R312C**	ccatgacgacacagtgctgtagctcttatctagca	tgctagataagagctacagcactgtgtcgtcatgg
**R312H**	catgacgacacagtgccatagctcttatctagcaa	ttgctagataagagctatggcactgtgtcgtcatg

**S314F**	acgacacagtgccgtagcttttatctagcaaatgaaatc	gatttcatttgctagataaaagctacggcactgtgtcgt
**Δ314-315**	cgacacagtgccgtagcctagcaaatgaaatcct	aggatttcatttgctaggctacggcactgtgtcg
**N318S**	ccgtagctcttatctagcaagtgaaatcctgtgggg	ccccacaggatttcacttgctagataagagctacgg
**W322C**	caaatgaaatcctgtgcggccaccgctatgagc	gctcatagcggtggccgcacaggatttcatttg
**R325C**	cctgtggggccactgctatgagcctgt	acaggctcatagcagtggccccacagg
**R325H**	ctgtggggccaccactatgagcctgtg	cacaggctcatagtggtggccccacag
**Y326N**	tggggccaccgcaatgagcctgtgc	gcacaggctcattgcggtggcccca
**E349K**	ggttccacaaaacttacaaagtccccaacactccc	gggagtgttggggactttgtaagttttgtggaacc
**P351S**	ccacaaaacttacgaagtcagcaacactcccctttgtagt	actacaaaggggagtgttgctgacttcgtaagttttgtgg
**C356F**	ccccaacactcccctttttagtgccagagactta	taagtctctggcactaaaaaggggagtgttgggg
**K364R**	gtgccagagacttagcagaaaggaaatatatcctctcaaatgc	gcatttgagaggatatatttcctttctgctaagtctctggcac
**S397N**	aaaatggagttccagaaaacactagtacggacacgc	gcgtgtccgtactagtgttttctggaactccatttt
**T400M**	tccagaaagcactagtatggacacgcccccct	agggggcgtgtccatactagtgctttctgga
**T402M**	gaaagcactagtacggacatgccccctgac	gtcagggggcatgtccgtactagtgctttc
**I406T**	gacacgccccctgacacggaccttcacaaccagg	cctggttgtgaaggtccgtgtcagggggcgtgtc
**P415L**	ccaggcaagtgtacttctagagcccaggc	gcctgggctctagaagtacacttgcctgg
**P420L**	ctagagcccaggctcttacggcgagag	ctctcgccgtaagagcctgggctctag

Table S3. Mutagenic primer pairs used to generate each Kir2.1 variant plasmid.

Kir2.1 variant	Flow total expression (% of WT)	Western expression (% of WT)	T_{agg} (°C)
WT	100	100 ± 18.2 (8)	59.2 ± 1 (13)
G52V	85.7 ± 15 (3)	16.4 ± 11.3 (5)*	50.6 ± 0.9 (3)*
R80C	NA	22.6 ± 11.4 (4)*	53.3 ± 2.9 (3)*
G100V	78.0 ±17.4 (6)	10.3 ± 4.7 (5)*	53.6 ± 2.8 (3)*
L109P	94.0 ± 19 (4)	34.1 ± 14.3 (8)*	55.7 ± 2 (6)*
**H110Y**	95.0 ± 17 (4)	59.8 ± 29.8 (7)*	57.6 ± 1.4 (5)
**C122Y**	73.9 ± 13.9 (6)	20.5 ± 29.6 (6)*	55.4 ± 2.0 (4)
**V123G**	106.0 ± 26.5 (4)	106.3 ±57.5 (6)	54.3 ± 2.8 (5)*
S136F	83.5 ± 13.7 (5)	23.0 ± 11.1 (5)*	54.3 ± 1.5 (3)*
T192I	91.5 ± 19 (6)	107.5 ± 49.3 (7)	56.1 ± 3.8 (5)
I269K	78.8 ± 17.5	23.8 ± 21.8 (4)*	55 ± 1.3 (3)*
L298R	90.3 ± 22 (3)	13.8 ± 13.1 (4)*	49.5 ± 1.4 (3)*
G300D	79 ± 18.7 (7)	77.1 ± 9.6 (4)	59.2 ± 4.8 (4)
**M307I**	81.6 ± 27.8 (6)	51.0 ±39.3 (4)*	59.9 ± 1.1 (3)
**R312H**	59.9 ± 17 (5)	4.7 ± 0.7 (3)*	61.7 ± 3.0 (3)
S314F	85 ± 25.2 (4)	16.5 ± 12.8 (3)*	50.6 ± 0.8 (3)*
∆314-315	74.3 ± 22.8 (5)	15.6 ±8.4(5)*	49.5 ± 0.7 (3)*
W322C	90.0 ± 16.4 (3)	51.7 ±16.4 (5)*	47.9 ± 2.4 (4)*
Y326N	94.7 ± 17.6 (3)	26.5 ± 19.5 (4)*	51.0 ± 1.1 (3)*
P351S	101 ± 20.5 (4)	88.1 ± 12.7 (3)	56.1 ± 2.2 (6)
T400M	83.0 ± 17.6 (4)	76.6 31.8 (3)	58.7 ± 0.2 (3)

Table S4. Kir2.1 variant total expression levels from flow cytometry and western blot analysis relative to WT as well as thermal melting temperature (T_{agg}) values. Variants in bold show no correlation between western blot expression (i.e. solubility) and Tagg. Asterisks indicate statistical significance ($P < 0.05$) using one-way ANOVA analysis with Dunett's post hoc test. Number of experiments in parentheses.

Kir2.1 variant	ClinVar	DMS function[5]	DMS surface[5]	LOF mechanism[7]	Surface changes
C54R	P	slight loss	slight loss	NA	NA
R67Q	P/LP	slight loss	slight loss	PIP2 binding	normal[8]
D71N	LP	slight gain	slight loss	PIP2 binding	normal[†]
D71V	P	slight loss	slight gain	NA	normal[9]
T75M	P	slight gain	slight loss	Mg2+ sensitivity	normal[8] loss[10]
T75R	P	little to no gain	slight loss	NA	normal[†]
D78G	P	slight gain	slight loss	PIP2 binding	normal[†]
D78N*	P	slight loss	slight gain	NA	NA
D78Y	P	NA	NA	PIP2 binding	normal[†]
R82Q	P/LP	slight gain	little to slight gain	PIP2 binding	normal[11,†]
R82W	P	slight gain	slight gain	PIP2 binding	normal[8,11]
L90R*	P	loss	loss	NA	NA
T142I*	LP	slight loss	slight loss	NA	NA
G144A	P	slight loss	slight loss	selectivity filter	NA
G144D	P	slight loss	gain	selectivity filter	loss[11]
G144S	P	loss	slight loss	selectivity filter	NA
Y145C	LP	slight gain	slight loss	selectivity filter	normal[12]
G146S	P	slight loss	NA	selectivity filter	normal[13]
D172N (SQTS)	P	NA	slight gain	NA	NA
P186G*	LP	NA	gain	NA	NA
P186Q	LP	slight loss	slight loss	PIP2 binding	NA
P186L	P	slight gain	loss	PIP2 binding	normal[†]
R189G*	LP	slight loss	slight loss	NA	NA
R189K*	LP	no change	slight loss	NA	NA
R189I	LP	little to no loss	slight loss	PIP2 binding	normal[†]
T192A	P	loss	slight loss	PIP2 binding	normal[†]
T192I	P	slight loss	little to slight gain	PIP2 binding	loss[†]
R213G*	LP	little to no gain	slight loss	NA	NA
G215D	P	slight loss	little to slight loss	PIP2 binding	normal[14]
N216Y*	LP	slight gain	slight loss	NA	NA
N216H	P	slight loss	slight loss	PIP2 binding	normal[9,†]
R218P	LP	slight gain	slight loss	NA	NA
R218Q	P	slight loss	slight loss	PIP2 binding	normal[9,†]
L225S	LP	slight gain	slight loss	NA	NA

R260H	LP	slight gain	slight loss	NA	NA
R260P	P	slight loss	little to slight loss	Trafficking	loss[15]
E299G*	LP	little to no gain	slight gain	NA	NA
G300D	P	no change	slight loss	PIP2 binding	loss[†]
G300V	P	no change	slight loss	PIP2 binding	loss[9]
M301K (SQTS)	P	slight gain	slight loss	NA	normal[†,16]
M301R*	P	slight gain	slight loss	NA	NA
V302M	P	slight gain	slight loss	PIP2 binding	loss[9,†]
T305A	LP	slight loss	slight loss	inward rectification	normal[8]
T305P	P	no change	slight loss	PIP2 binding	NA
M307V	P/LP	slight gain	slight loss	PIP2 binding	normal[17]
T309I	P	slight gain	little to slight loss	PIP2 binding	normal[18]
R312C	P/LP	slight gain	loss	PIP2 binding	normal[†]
R312H	P/LP	slight loss	slight loss	PIP2 binding	loss[†] normal[19]

Table S5. All Kir2.1 variants classified as pathogenic (P) and likely pathogenic (LP) in ClinVar with qualitative descriptions of their function and surface expression from a DMS[6]. LOF mechanism descriptions obtained from review by Moreno-Manuel and co-authors[7]. Some mechanisms inferred only from location. Shaded variants disagree with DMS functional data[5] (i.e. no LOF or GOF). Asterisks indicate no functional data has been reported to the best of our knowledge. Numbers reference sources for the surface expression descriptions. Dagger indicates this study. NA is not available.

Kir2.1 variant	ClinVar	-50mV (pA/pF)	-100mV (pA/pF)
**WT**	NA	1.89 ± 0.34 (11)	-37.5 ± 4.3 (11)
**D71Y**	VUS	-0.41 ± 0.33 (7)	-0.46 ± 0.34 (7)
**V123G**	VUS	0.33 ± 0.23 (8)	0.47 ± 0.61 (8)
**S136F**	VUS	-0.94 ± 0.28 (9)	-1.90 ± 0.67 (9)
**P186L**	P	0.44 ± 0.89 (7)	3.55 ± 1.94 (7)
**K187R**	VUS	0.03 ± 0.19 (8)	-0.63 ± 0.61 (8)
**T192I**	VUS	0.19 ± 0.75 (9)	1.10 ± 1.39 (9)
**G206S**	VUS	0.88 ± 0.32 (7)	-19.8 ± 2.9 (7)
**L298R**	VUS	-0.21 ± 0.41 (8)	-0.21 ± 0.54 (8)
**M307I**	VUS	-0.96 ± 0.90 (9)	-1.29 ± 1.25 (9)
**R312C**	P/LP	-0.31 ± 0.45 (8)	-0.31 ± 0.93 (8)
**R312H**	P/LP	-0.54 ± 0.25 (9)	-0.97 ± 0.50 (9)
**P351S**	VUS	4.17 ± 1.17 (11)	-68.4 ± 7.0 (11)
**T400M**	VUS	0.59 ± 0.23 (8)	-5.95 ± 2.12 (8)

Table S6. Current densities of ATS-linked Kir2.1 variants. Mean ± SD current at -50mV and -100mV for each variant. N in parentheses.

Supplementary References

1. Marrus, S. B., Cuculich, P. S., Wang, W. and Nerbonne, J. M. (2011). Characterization of a novel, dominant negative KCNJ2 mutation associated with Andersen-Tawil syndrome. *Channels* 5, 500–509.

2. Verma, K., Shanthi, V. and Ramanathan, K. (2015). Investigation of Missense Mutations in KCNJ2 Gene: A Computational Approach. *Res. J. Pharm. Technol.* 8, 1540.

3. Delannoy, E., Sacher, F., Maury, P., Mabo, P., Mansourati, J., Magnin, I., Camous, J.-P., Tournant, G., Rendu, E., Kyndt, F., et al. (2013). Cardiac characteristics and long-term outcome in Andersen–Tawil syndrome patients related to KCNJ2 mutation. *Europace* 15, 1805–1811.

4. Limberg, M. M., Zumhagen, S., Netter, M. F., Coffey, A. J., Grace, A., Rogers, J., Böckelmann, D., Rinné, S., Stallmeyer, B., Decher, N., et al. (2013). Non dominant-negative KCNJ2 gene mutations leading to Andersen-Tawil syndrome with an isolated cardiac phenotype. *Basic Res. Cardiol.* 108, 353.

5. Macdonald, C. B., Nedrud, D., Grimes, P. R., Trinidad, D., Fraser, J. S. and Coyote-Maestas, W. (2023). DIMPLE: deep insertion, deletion, and missense mutation libraries for exploring protein variation in evolution, disease, and biology. *Genome Biol.* 24, 36.

6. Coyote-Maestas, W., Nedrud, D., He, Y. and Schmidt, D. (2022). Determinants of trafficking, conduction, and disease within a K+ channel revealed through multiparametric deep mutational scanning. *eLife* 11, e76903.

7. Moreno-Manuel, A. I., Gutiérrez, L. K., Vera-Pedrosa, M. L., Cruz, F. M., Bermúdez-Jiménez, F. J., Martínez-Carrascoso, I., Sánchez-Pérez, P., Macías, Á. and Jalife, J. (2022). Molecular stratification of arrhythmogenic mechanisms in the Andersen Tawil syndrome. *Cardiovasc. Res.* 119, 919–932.

8. Eckhardt, L. L., Farley, A. L., Rodriguez, E., Ruwaldt, K., Hammill, D., Tester, D. J., Ackerman, M. J. and Makielski, J. C. (2007). KCNJ2 mutations in arrhythmia patients referred for LQT testing: A mutation T305A with novel effect on rectification properties. *Heart Rhythm* 4, 323–329.

9. Bendahhou, S., Donaldson, M. R., Plaster, N. M., Tristani-Firouzi, M., Fu, Y.-H. and Ptácek, L. J. (2003). Defective Potassium Channel Kir2.1 Trafficking Underlies Andersen-Tawil Syndrome*. *J. Biol. Chem.* 278, 51779–51785.

10. Tani, Y., Miura, D., Kurokawa, J., Nakamura, K., Ouchida, M., Shimizu, K., Ohe, T. and Furukawa, T. (2007). T75M-KCNJ2 mutation causing Andersen–Tawil syndrome

enhances inward rectification by changing Mg2+ sensitivity. *J. Mol. Cell. Cardiol.* 43, 187–196.

11. Kimura, H., Zhou, J., Kawamura, M., Itoh, H., Mizusawa, Y., Ding, W.-G., Wu, J., Ohno, S., Makiyama, T., Miyamoto, A., et al. (2012). Phenotype Variability in Patients Carrying KCNJ2 Mutations. *Circ.: Cardiovasc. Genet.* 5, 344–353.

12. Scheiper, S., Hertel, B., Beckmann, B.-M., Kääb, S., Thiel, G. and Kauferstein, S. (2017). Characterization of a novel KCNJ2 sequence variant detected in Andersen-Tawil syndrome patients. *BMC Méd. Genet.* 18, 113.

13. Haruna, Y., Kobori, A., Makiyama, T., Yoshida, H., Akao, M., Doi, T., Tsuji, K., Ono, S., Nishio, Y., Shimizu, W., et al. (2007). Genotype-phenotype correlations of KCNJ2 mutations in Japanese patients with Andersen-Tawil syndrome. *Hum. Mutat.* 28, 208–208.

14. Hosaka, Y., Hanawa, H., Washizuka, T., Chinushi, M., Yamashita, F., Yoshida, T., Komura, S., Watanabe, H. and Aizawa, Y. (2003). Function, subcellular localization and assembly of a novel mutation of KCNJ2 in Andersen's syndrome. *J. Mol. Cell. Cardiol.* 35, 409–415.

15. Barajas-Martinez, H., Hu, D., Ontiveros, G., Caceres, G., Desai, M., Burashnikov, E., Scaglione, J. and Antzelevitch, C. (2010). Biophysical and Molecular Characterization of a Novel De Novo KCNJ2 Mutation Associated With Andersen-Tawil Syndrome and Catecholaminergic Polymorphic Ventricular Tachycardia Mimicry. *Circ.: Cardiovasc. Genet.* 4, 51–57.

16. Hattori, T., Makiyama, T., Akao, M., Ehara, E., Ohno, S., Iguchi, M., Nishio, Y., Sasaki, K., Itoh, H., Yokode, M., et al. (2012). A novel gain-of-function KCNJ2 mutation associated with short-QT syndrome impairs inward rectification of Kir2.1 currents. *Cardiovasc. Res.* 93, 666–673.

17. Handklo-Jamal, R., Meisel, E., Yakubovich, D., Vysochek, L., Beinart, R., Glikson, M., McMullen, J. R., Dascal, N., Nof, E. and Oz, S. (2020). Andersen–Tawil Syndrome Is Associated With Impaired PIP2 Regulation of the Potassium Channel Kir2.1. *Front. Pharmacol.* 11, 672.

18. Bendahhou, S., Fournier, E., Sternberg, D., Bassez, G., Furby, A., Sereni, C., Donaldson, M. R., Larroque, M., Fontaine, B. and Barhanin, J. (2005). In vivo and in vitro functional characterization of Andersen's syndrome mutations. *J. Physiol.* 565, 731–741.

19. Zuniga, D., Zoumpoulakis, A., Veloso, R. F., Peverini, L., Shi, S., Pozza, A., Kugler, V., Bonneté, F., Bouceba, T., Wagner, R., et al. (2024). Biochemical, biophysical, and structural investigations of two mutants (C154Y and R312H) of the human Kir2.1 channel involved in the Andersen-Tawil syndrome. *FASEB J.* 38, e70146
